# New geochemical and Sr-Nd-Hf isotopic constraints on the genesis of kimberlites and ultramafic lamprophyres from the Adelaide Fold Belt and Gawler Craton, South Australia

**DOI:** 10.1007/s00710-025-00938-w

**Published:** 2025-07-09

**Authors:** Hayden Dalton, Andrea Giuliani, Angus Fitzpayne, Bradley J. Peters

**Affiliations:** 1 School of Geography, Earth and Atmospheric Sciences, The University of Melbourne, Parkville, Victoria 3010 Australia; 2 Earth and Planets Laboratory, Carnegie Institution for Science, Washington, 20015 DC USA; 3 Department of Earth and Planetary Sciences, Institute of Geochemistry and Petrology, ETH Zurich, 8092 Zurich, Switzerland

**Keywords:** Kimberlite, Ultramafic lamprophyre, Isotope geochemistry, Crustal contamination, Chemical geodynamics, South Australia

## Abstract

This study investigates the geochemical and isotopic characteristics of Jurassic kimberlites and ultramafic lamprophyres (UMLs) from four clusters within the Adelaide Fold Belt (AFB) and two within the Gawler Craton in South Australia. Petrographic analysis, including the occurrence of magmatic clinopyroxene in the groundmass, supported by a review of available mica and spinel compositional data, indicates that many previously classified kimberlites (Eurelia, Angaston and Terowie) are, in fact, ultramafic lamprophyres. New whole-rock major-, trace element and Sr-Nd-Hf isotopic results, augmented by in-situ perovskite and carbonate Sr isotopes, reveal that this sample suite exhibits extensive geochemical variability. These new data highlight the significant role of crustal contamination in modifying not only bulk-rock major, trace element and Sr isotope systematics, the latter being pristine exclusively in low-SiO_2_ samples, but also Nd and Hf isotopic signatures. This is most evident for the Mount Hope (εNd_(i)_ = -5.1 to -1.5; εHf_(i)_ = -8.6 to -2.7) and Cleve (εNd_(i)_ = -3.7 to -0.2; εHf_(i)_ = -6.6 to +0.3) kimberlites of the Gawler Craton which display geochemically enriched compositions that are rarely seen in kimberlitic rocks. In contrast, the AFB samples exhibit less inter-sample isotopic variability and have compositions that are more typical of kimberlites and UMLs globally (εNd_(i)_ = +0.3 to +3.9; εHf_(i)_ = +0.7 to +6.6). There is no clear lithospheric thickness control governing the absence of UMLs on the Gawler Craton and their presence within the AFB. Similarly, there are no systematic differences in Sr–Nd-Hf isotopes between uncontaminated kimberlites and UMLs, arguing against obvious differences in their asthenospheric sources. We tentatively suggest that contribution by more pervasively metasomatised lithospheric mantle beneath the AFB compared to the Gawler craton (based on existing garnet xenocryst data) may facilitate the formation of ultramafic lamprophyres in this region. While subduction along the southern palaeo-margin of Pangea likely did not directly trigger magmatism, it may have facilitated deep mantle upwelling linked to the contemporaneous Karoo-Ferrar Large Igneous Province, with related formation of kimberlites and UMLs in South Australia.

**Supplementary Information:**

The online version contains supplementary material available at 10.1007/s00710-025-00938-w.

## Introduction

Kimberlites are enigmatic, hybrid, small-volume ultramafic igneous rocks that have intruded through predominantly ancient cratonic mantle lithosphere since 2.0 Ga or perhaps 2.8 Ga (e.g., Mitchell 1986; Giuliani and Pearson 2019; Heaman et al. 2019). These rocks are the primary conveyer of diamonds to the Earth’s surface and represent some of the deepest derived mantle melts on the planet (e.g., Haggerty 1994; De Wit 2010; Stamm and Schmidt 2018; Pearson et al. 2019; Giuliani et al. 2023). The observation that kimberlites do not always occur in isolation, and are instead readily found associated with a diversity of other ultramafic to alkaline rock types such as olivine lamproites, ultramafic lamprophyres and carbonatites, is becoming increasingly recognised (e.g., Tappe et al. 2008; Nielsen et al. 2009; Dalton et al. 2019; Sarkar et al. 2021; Schmidt et al. 2024). Even where these rocks are emplaced broadly coevally, it remains an open question as to the presence of a genetic relationship and whether they may sit together on a compositional continuum. Another outstanding question in the study of kimberlites is the drivers of their geochemical variation, particularly as it relates to their isotopic compositions. A growing body of evidence suggests that subducted material may play a pivotal role in diversifying the geochemical signatures of kimberlites (Nowell 2004; Tappe et al. 2013; Woodhead et al. 2019; Xu et al. 2021; Fitzpayne et al. 2021; Dalton et al. 2022; Giuliani et al. 2022, 2025; van Blerk et al. 2025). More specifically, Woodhead et al. (2019) suggested that Mesozoic kimberlites located along the western paleo-margin of the supercontinent Pangea including those from western Canada, Brazil and southern Africa, were all affected by a deep subduction signature, which was absent in kimberlites further east (e.g., southwestern Greenland, Siberia). An outstanding question is whether kimberlites from the southern margin of Pangea were similarly impacted by subduction of oceanic lithosphere in their deep mantle source.

The kimberlites and related rocks of South Australia may be used as a case study to address some of these unresolved questions. The South Australian craton hosts a number of clusters of broadly coeval kimberlites, some of which are reclassified here as ultramafic lamprophyres, hence providing the opportunity to address the origin of these distinct magmas within the same restricted geodynamic context. Furthermore, these rocks were emplaced on the southern margin of Gondwana and may therefore yield insights into the occurrence of a subduction signature in the source region of kimberlites that were relatively proximal to the convergent margins of Pangea.

The occurrence of ‘kimberlites’ in South Australia has been documented since the 1970s (e.g., Colchester 1972; Ferguson and Sheraton 1977, 1982; Ferguson 1980; Stracke et al. 1982; Scott-Smith et al. 1984; Wyatt et al. 1994; Cooper and Morris 2012) following systematic exploration by De Beers, although alluvial diamonds were discovered by gold prospectors in the nineteenth century. Despite this history, and the subsequent discovery of over 200 occurrences of kimberlites and similar ultramafic rock types, relatively little is known about the petrogenesis and mantle source of these bodies. Notable exceptions include the petrological, mineral chemical and geochronological study on samples from the Adelaide Fold Belt (AFB) of Tappert et al. (2019), who also provided an initial set of Sr–Nd isotope analyses for perovskite separates. In addition, Sudholz et al. (2022) provided an up-to-date assessment of the lithological and pressure–temperature conditions of the lithospheric mantle entrained by the South Australian kimberlites and related rocks.

**Fig. 1:**
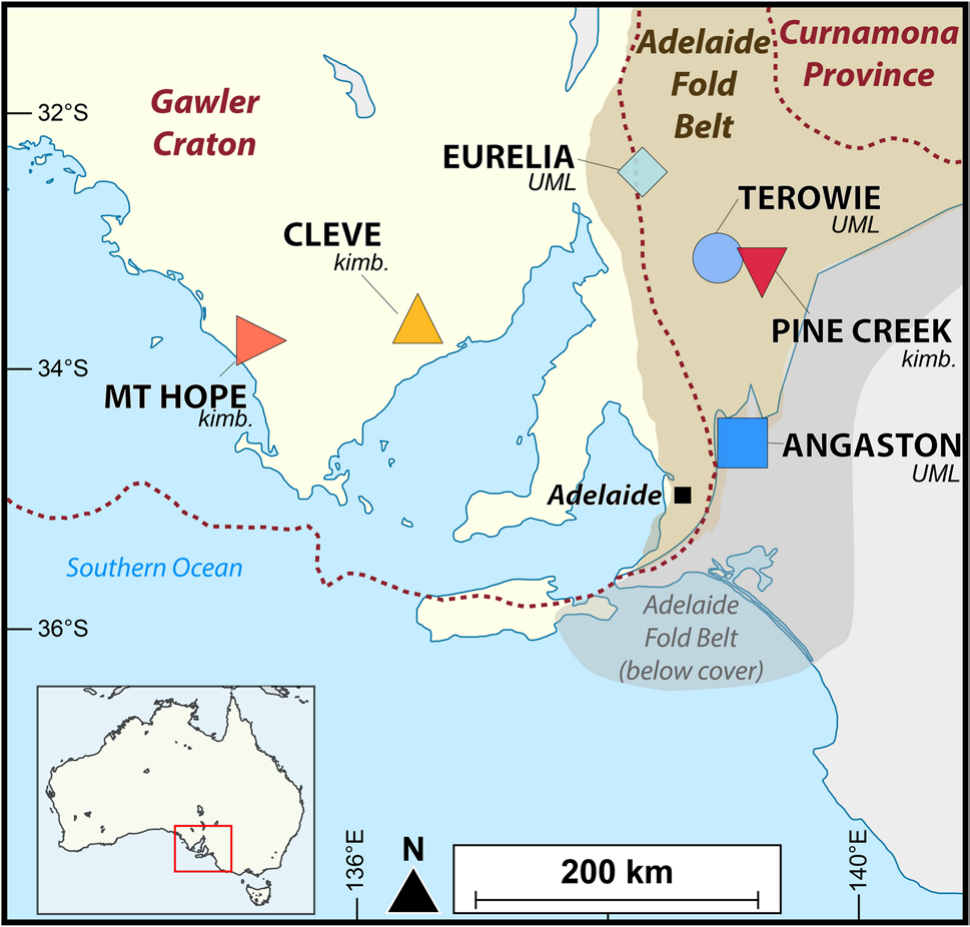
Map showing location of studied kimberlites (kimb.) and ultramafic lamprophyres (UML) in the Adelaide Fold Belt and Gawler Craton, South Australia (modified after Cooper and Morris 2012)

Here, we present new petrographic and whole-rock geochemical data for Jurassic kimberlites (Cleve, Mount Hope and Pine Creek) and ultramafic lamprophyres (UMLs; Angaston, Terowie and Eurelia) from South Australia that have intruded through either the Late Archean to Mesoproterozoic Gawler Craton (Cleve and Mount Hope) or the Neoproterozoic to Cambrian Adelaide fold-belt (the remaining clusters; Fig. 1). We also report the first bulk-rock Hf isotopic compositions for this suite of rocks, alongside new whole-rock Sr–Nd isotopes and in situ Sr isotope data for perovskite and carbonate.

## Geological setting and samples

The South Australian Craton is a ‘composite’ craton including the Coompana Block, the Gawler Craton and the Curnamona Province, with the latter two nuclei separated by the AFB (Fig. 1). The lithospheric structure beneath South Australia has been examined using kimberlite-borne xenoliths with thickness estimates ranging between 150–180 km (Scott-Smith et al. 1984; Gaul et al. 2003; Tappert et al. 2011, 2019). More recently, Sudholz et al. (2022) provided a comprehensive appraisal of the chemo-lithostratigraphy underlying the South Australian Craton, revealing a complex, heterogeneously metasomatised subcontinental lithospheric mantle with an undulating lithosphere-asthenosphere boundary that locally exceeds 200 km depth. Kimberlites and related rocks have been predominantly emplaced in the AFB, with fewer localities also hosted in the Gawler Craton. The majority of this activity occurred during the Jurassic (~170–190 Ma; Stracke et al. 1982; Wyatt et al. 1994; Cooper and Morris 2012; Tappert et al. 2019) although Ordovician ‘lamprophyres’ have also been documented along the eastern margin of the AFB (Jaques and Milligan 2004; Jaques 2006).

As is typical of these magmas in localities worldwide, kimberlites and related rocks in South Australia have been emplaced in discrete ‘clusters’ (or larger and longer-lived ‘fields’). The most notable ones occur at Elliston/Mount Hope, Cleve and Port Augusta on the Gawler Craton, with Eurelia (also referred to as Orroroo), Truro and Terowie hosted inside the AFB, where over 100 kimberlite and related intrusions are located (Cooper and Morris 2012). This study focuses on samples (*n* = 20) from six Jurassic clusters: Mount Hope (191 ± 17 Ma; U–Pb perovskite; Cooper and Morris 2012), Cleve (180 ± 3 Ma; U–Pb perovskite; Wyatt et al. 1994), Eurelia (170 ± 2 Ma; U–Pb zircon; Scott-Smith et al. 1984; Black et al. 1993), Angaston of the Truro field (181 ± 3 Ma; U–Pb perovskite Ma; Tappert et al. 2019), as well as the Pine Creek (188 Ma; Rb–Sr phlogopite-whole rock; Cooper and Morris 2012) and Terowie (~196 ± 2 Ma; U–Pb perovskite; Tappert et al. 2019) clusters of the Terowie field.

## Methods

### Sample selection and preparation

The material from this study was sourced from Stockdale Prospecting Ltd (i.e. the subsidiary of De Beers Exploration in Australia during the 1980s and 1990s) and the South Australia Drill Core Reference Library. A sub-set of the available collection was employed for this work with sample selection based on wide geographic coverage and extent of alteration. Relatively fresh material was available only for some localities, and where not available we included samples which preserved part of the original magmatic mineralogy and particularly perovskite, which is the main host of incompatible trace elements (including Nd and Hf) in kimberlites and related rocks.

For each sample, rock fragments and segments of drill-core were selected based on the absence of visible crustal or mantle xenoliths. A small portion of this material was utilised to make petrographic thin sections which were inspected using a polarised light microscope. Additional material from these selected samples were crushed using a steel jaw crusher. Small chips (< 1 cm) representing the freshest available material, devoid of obvious crustal xenoliths/xenocrysts, were selected under a binocular microscope and then powdered using an agate mill for whole-rock major-, trace-element and isotopic analyses using the methods described by Fitzpayne et al. (2023). As it will be shown below, this approach did not prevent crustal contamination of some of the analysed samples due to contribution of small crustal fragments in the volcaniclastic kimberlites.

### Major element analysis by X-ray fluorescence (XRF)

Approximately 1 g of each powdered sample was placed into a ceramic crucible. The total loss on ignition (LOI) was determined in an oxygen atmosphere at 500 °C. Following this, the sample was blended in a 1:7 ratio with a lithium tetraborate flux. The mixture was then heated in a platinum crucible at 1000 °C to create a circular glass disc for subsequent XRF measurements, using a wavelength-dispersive X-ray spectrometer (WDS) Axios system from Malvern Panalytical. A range of certified international and synthetic standards was employed for primary calibration and the natural basalt BCR-2 was measured as a secondary reference material to check accuracy.

To measure C contents of the same sample powders, approximately 5 mg of each powder was mixed with 1 g of Cu chips in a ceramic crucible. The combined samples were combusted in an oxygen-rich environment and C contents were measured by infrared absorption using a LECO CS844 analyser relative to LECO reference material 502–950 (synthetic carbon).

### Trace element analysis

Approximately 100 mg of the same sample powders described above were dissolved in 3:1 HF-HNO_3_ for 48 h on a hotplate. The resulting solutions were dried down and repeatedly dosed with concentrated HNO_3_, after which the samples were dissolved in ∼5 M HNO_3_. The resulting solutions were generally clear and free of solid residue.

A 10% fraction of the dissolved sample was set aside for trace element analysis and dried down before dilution into 2% HNO_3_. Trace element concentrations were obtained following the procedures of Eggins et al. (1997) and Kamber et al. (2003). A multi-element spike containing In and Bi was added prior to analysis of the solutions, which was undertaken using an Element XR sector field inductively coupled plasma-mass spectrometry (ICP-MS) system. The isotopes ^115^In and ^209^Bi were used as internal standards, and the CRPG basalt reference material BE-N was used for calibration. Internal counting statistics for multiple runs of the same sample solutions show that uncertainties for all elements are < 5%. USGS basalt BCR-2 was analysed as unknowns for quality control and yielded results consistent with published reference values (Table S5).

### Radiogenic isotope analysis

The remaining solutions (i.e., those not employed for trace element analyses) were dried down, and Sr, Nd, and Hf were separated therefrom using ion-exchange column-chromatography methods adapted from Münker et al. (2001) and Pin et al. (2014). Separated Sr fractions were first treated with 6 M HCl, repeatedly adding several drops at a time and evaporated at 80 °C. The samples were then dissolved in 6 M HCl and left to equilibrate for an hour. Once dissolved, the samples were pipetted onto Re single filaments along with an equal volume of a Ta_2_O_5_ activator. Strontium isotope analyses were carried out using a Thermo-Fisher Triton thermal ionisation mass spectrometer (TIMS). Instrumental mass bias was corrected by internal normalization to ^88^Sr/^86^Sr = 8.37521 using the exponential law, and ^87^Sr/^86^Sr additionally normalised to a preferred ^87^Sr/^86^Sr ratio of 0.710249 for the NBS SRM987 standard based on the average measured ^87^Sr/^86^Sr ratio for SRM987 in the barrel in which the sample was analysed. Several international reference materials (USGS basalts BCR-2 and BHVO-2; kimberlite SARM-39) were analysed as unknowns alongside the kimberlite samples, and returned values within uncertainty of expected values (Table S5 in the electronic supplementary material; ESM).

Isotopic analyses of Nd and Hf were carried out using a Nu Plasma II multi-collector (MC) ICP-MS system. Samples and standards were diluted to ensure total Nd and Hf signals were between 15 and 20 V, and within 10% of the solution standards. Instrumental mass bias was corrected by normalization to ^146^Nd/^144^Nd = 0.7219 and ^179^Hf/^177^Hf = 0.7325 using the exponential law. ^143^Nd/^144^Nd and ^176^Hf/^177^Hf ratios for unknowns and secondary standards are normalised to La Jolla Nd = 0.511858 (Jweda et al. 2016) and JMC475 = 0.282160 (Vervoort and Blichert-Toft 1999), respectively. External precisions (2*s*) are ± 0.000016 for ^143^Nd/^144^Nd and ± 0.000012 for ^176^Hf/^177^Hf, based on the long-term reproducibility of standard reference materials in this laboratory (Table S5), including the J-Nd solution standard (0.512114 ± 0.000016; n = 68; compared to the accepted value of 0.512099 (Garçon et al. 2018; Table S5). Age corrections were calculated using the published emplacement ages described above and ^87^Rb/^86^Sr, ^147^Sm/^144^Nd and ^176^Lu/^177^Hf ratios derived from trace element data for the same sample solutions (Tables S1, S2). εNd and εHf values are calculated relative to the chondritic (CHUR) composition of Bouvier et al. (2008). The Rb, Sm and Lu decay constants are 1.397 × 10^−11^ yr^−1^, 6.54 × 10^−12^ yr^−1^ and 1.865 × 10^−11^ yr^−1^, respectively.

### In situ laser ablation MC-ICP-MS analysis of Sr isotopes

In situ Sr isotope analyses of perovskite and carbonate in thin section were undertaken using a RESOlution 193 nm excimer laser probe interfaced to the same Nu Plasma II MC-ICPMS employed for the solution-mode isotopic analyses and following similar methods as Fitzpayne et al. (2023) and Tavazzani et al. (2024). Analytical conditions included a pulse rate of 5 Hz, 30 to 50 μm spot size and laser fluence of ∼4 J/cm^2^ for perovskite, and similar to larger spot size (50 to 80 μm) and ∼2 J/cm^2^ of laser fluence for carbonates. Each analysis consisted of a sequence of 30 s of background measurement, 50 s of ablation time and 30 s for washout. Data reduction, including corrections for isobaric interferences (Kr, Ca dimers, Ca argides, Rb, and, for perovskite only, doubly-charged rare earth elements, REE) and instrumental mass bias was performed using the Iolite software (Paton et al. 2011, 2007b). Krypton was removed by on-peak subtraction whereas Ca dimers, Ca argides, Rb and doubly-charged REE were evaluated by monitoring the Sr-free masses and half-masses 82, 83, 83.5, 85 and 86.5. Instrumental drift was evaluated by repeated measurements of perovskite 83P13 (Paton et al. 2007b) and carbonate MMC (Modern Marine Carbonate; Woodhead et al. 2005) and the perovskite and carbonate data are reported relative to 83P13 ^87^Sr/^86^Sr of 0.72500 and MMC ^87^Sr/^86^Sr of 0.70916, respectively, via standard bracketing (see Tables S3 and S4 for complete set of analyses). Analytical accuracy was evaluated by repeated ablation of Ice River IR-6 (D Graham Pearson, unpublished data), and carbonate MW2 (in-house standard), which returned weighted mean ^87^Sr/^86^Sr consistent with isotope-dilution values (see Tables S2-S4). Mean ^84^Sr/^86^Sr of both reference materials and unknowns are within uncertainty of the nominal ratio (0.0565). ^87^Rb/^86^Sr ratios are generally less to much less than 0.01, which makes corrections for ^87^Sr/^86^Sr ingrowth insignificant. Therefore, the reported Sr isotope ratios are considered to be equal to the isotope ratios at time of emplacement. Note that a final screen was applied to the data on the basis of ^87^Rb/^86^Sr (acceptable range ≤ 0.01) and ^84^Sr/^86^Sr ratios (acceptable range 0.05 to 0.06).

## Results

### Petrography

Many of the samples in the present study are variably altered and as such the below descriptions will focus on the notable diagnostic features of each intrusion/body (see Table 1 for a summary). Although all the examined localities were previously considered to be kimberlites (Scott-Smith et al. 1984; Wyatt et al. 1994; Tappert et al. 2019), our petrographic re-evaluation paired with available mineral chemical data (Scott-Smith et al. 1984; Wyatt et al. 1994; Tappert et al. 2019) indicates that most of the examined samples from the Adelaide Fold Belt (AFB) rocks are ultramafic lamprophyres – see details and discussion section further below.

**Table 1:** Petrographic description and classification of samples from Gawler Craton and Adelaide Fold Belt, South Australia

Locality	Sample number(s)	Coherent or volcaniclastic	Mineralogy/features	Classification
Cleve-01	CL-6a	Volcaniclastic	Distinct clasts containing pseudomorphs (serpentine ± carbonate) of olivine macrocrysts, perovskite and spinel + common crustal lithics	Kimberlite
	CL-6b, CL-6c	Coherent, aphanitic	Pseudomorphs (serpentine ± carbonate) of olivine microcrysts (< 1 mm), abundant perovskite and spinel. Pervasive alteration of the serpentine-dominated groundmass with minor carbonate	Kimberlite
Mount Hope-01, −04 and −05	MH1 DH55Z, MH14 (SH18 DH15), SH14 DH021	Volcaniclastic	Dominated by spheroid/rounded magmaclasts which preserve fresh perovskite. Also present are pseudomorphs (serpentine) of olivine macrocrysts, spinel grains and crustal lithics occasionally coring magmaclasts. Interclast serpentine	Kimberlite
Mount Hope-02	MH2 DH56 J	Volcaniclastic	Pseudomorphs (serpentine) of olivine macrocrysts. Highly altered, oxide minerals absent (spinel, perovskite etc.). Crustal lithics	Kimberlite
Pine Creek	BDDH1b	Coherent, macrocrystic	Pseudomorphs (serpentine) of olivine macrocrysts and microcrysts, atoll spinel, large perovskite (> 100 µm) and colourless mica (possibly kinoshitalite-phlogopite) in the groundmass. Carbonate and lesser serpentine in the mesostasis	Kimberlite
	BDDH1a	Coherent, locally banded	Dominant carbonate (> 50 vol%) with abundant opaque (probably spinel) and minor serpentine	Carbonatite
	BURRA B76	Coherent, macrocrystic	Olivine macrocrysts and primary groundmass mineralogy predominantly replaced by carbonate and minor serpentine with spinel remaining	Kimberlite (?)
Angaston-01	DD92 TR1b	Coherent, porphyritic	Serpentine pseudomorphs after olivine microcrysts and lesser macrocrysts, minor phlogopite microcrysts, groundmass consisting of phlogopite, clinopyroxene, spinel, perovskite and interstitial carbonates which also occur in isolated ‘pockets’	Ultramafic lamprophyre
	DD92 TR1a,1c	Coherent, porphyritic	Pervasively carbonatised versions of sample 1b. Clinopyroxene is not observed, olivine is replaced by carbonate. Serpentine is very scarce in 1a, perovskite is absent in 1c	Ultramafic lamprophyre (?)
Eurelia	CD10c	Coherent, locally porphyritic	Carbonate pseudomorphs after olivine microcrysts, groundmass of phlogopite, clinopyroxene, spinel, altered perovskite, interstitial carbonate and serpentine	Ultramafic lamprophyre
	CD10b	Coherent, aphanitic	Microphenocrysts of phlogopite and clinopyroxene with interstitial carbonate and lesser spinel	Ultramafic lamprophyre
	CD10a	Coherent	Very altered sample with serpentinised olivine macrocrysts and altered mica macrocrysts and microcrysts plus spinel and altered perovskite	Kimberlite or ultramafic lamprophyre (unclear)
Terowie 6	TSDD2a, TSDD2b	Coherent, porphyritic	Serpentine pseudomorphs after olivine macrocrysts and microcrysts, abundant and locally poikilitic red-brown phlogopite macrocrysts and phenocrysts with tetraferriphlogopite rims, fine-grained spinel, clinopyroxene plus interstitial carbonate and serpentine	Ultramafic lamprophyre
	TSDD3a, TSDD3b	Coherent, porphyritic	As above with carbonate replacing olivine and less abundant phlogopite	Ultramafic lamprophyre

Note: Where samples display similar features they have been grouped together

#### Kimberlites

**Fig. 2:**
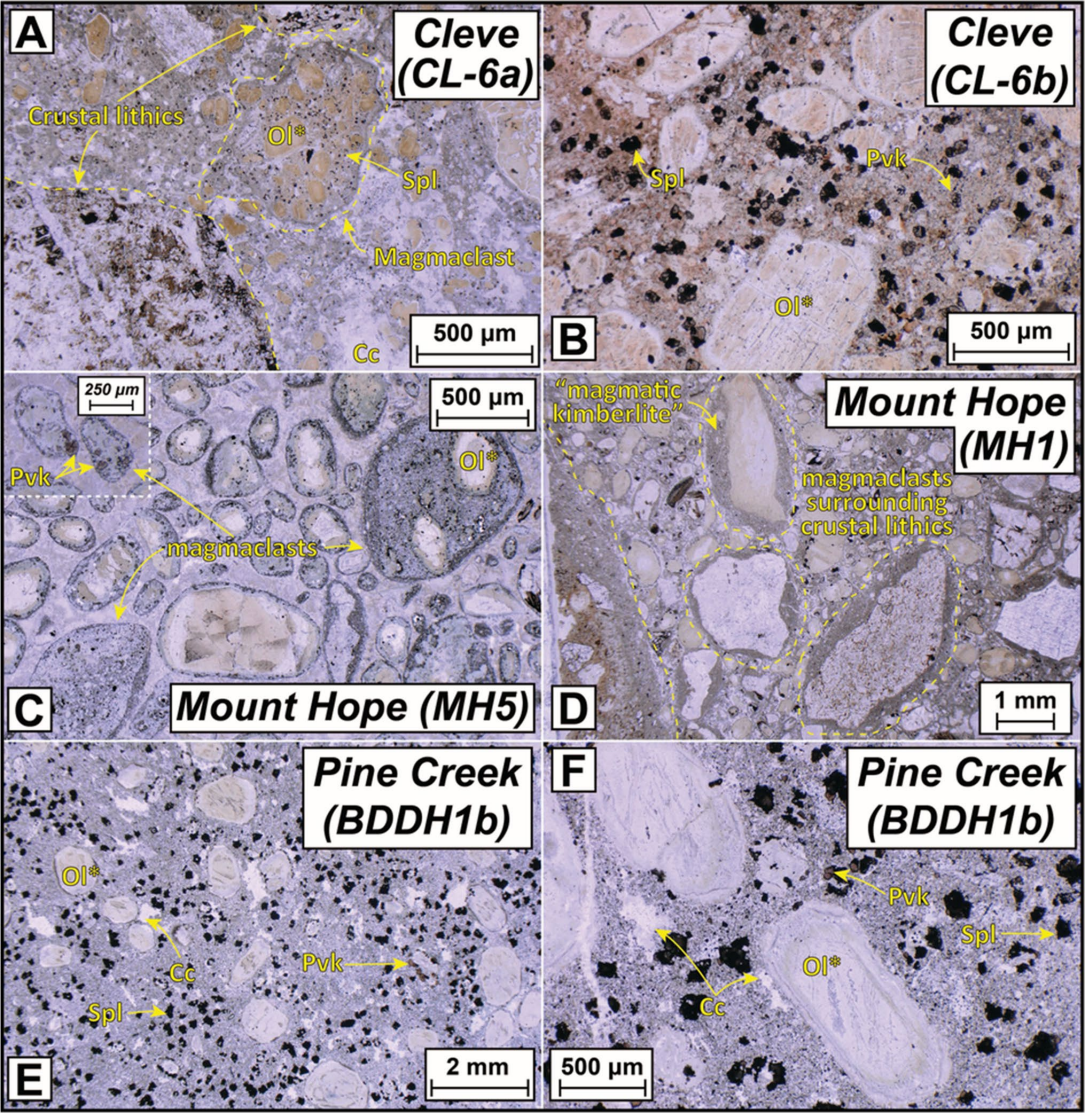
Representative plane-polarised transmitted-light photomicrographs for the three kimberlite localities sampled for this study: **A** and **B**) Cleve; **C** and **D**) Mount Hope; **D** and **E**) Pine Creek. Abbreviations of mineral names: Spl – spinel; Pvk – perovskite; Ol* – pseudomorph after olivine; Cc – calcite. See Table 1 for detailed descriptions

The samples from Cleve (pipe Cleve-01) include a volcaniclastic (CL6a) and two coherent aphanitic (CL6b, 6c), altered kimberlites. The former includes magmaclasts with one or more macrocrysts and microcrysts (> 1 and < 1 mm, respectively) of serpentinised olivine and small grains of spinel and perovskite in a serpentine-rich matrix, common lithic fragments and an interclast matrix of carbonate, serpentine and other unrecognised low-temperature silicate minerals (Fig. 2A). The coherent kimberlite samples show an inequigranular texture dominated by pseudomorphs (serpentine ± carbonate) of olivine microcrysts (Fig. 2B). The groundmass contains abundant perovskite and spinel (~ 50–100 µm) which in places form a ‘necklace’ around the olivine. The mesostasis is dominated by serpentine with carbonate segregations and cross-cutting veinlets of carbonate also present.

The four samples from Mount Hope represent four pipes: Mount Hope-01, -02, -04 and -05. They are all composed of altered volcaniclastic kimberlite. As with Cleve, olivine macrocrysts have been replaced by serpentine and carbonate. A striking feature of the Mount Hope samples is the abundance of rounded magmaclasts cored by pseudomorphed olivine macrocrysts (Fig. 2C) or lithic fragments (Fig. 2D), within which fresh perovskite is preserved (magmaclasts are fluidal-shaped bodies of kimberlite magma (now solidified) formed by any process of magma disruption prior to solidification; Webb and Hetman 2021). Serpentine is the dominant interclast phase.

Of the three samples from Pine Creek (locality Pine Creek-04) one (BDDH1b), is an altered coherent macrocrystic kimberlite with prominent serpentinised olivine macrocrysts, tablet-shaped olivine microcrysts and abundant ‘atoll’ spinels and perovskite (> 100 µm) with colourless mica micro-phenocrysts (likely kinoshitalite-phlogopite solid solution), carbonate dominates the interstices between these phases with lesser serpentine (Fig. 2E,F). A second sample (BDDH1a) is a spinel-bearing carbonatite, dominated by carbonate (> 50 vol%) with lesser amounts of an oxide phase, likely spinel, and minor serpentine (Figure S1 in the ESM). Based on the geographic association with kimberlite sample BDDH1b and, as shown below, indistinguishable isotopic composition of these two samples, we consider BDDH1a to be a carbonatite cumulate or, perhaps segregate related to kimberlite melts (e.g., similar to Benfontein—Abersteiner et al. 2019; Dawson and Hawthorne 1973). A third sample from Pine Creek (BURRA B76) exhibits a coherent macrocrystic texture and features serpentinised olivine macrocrysts and a totally altered groundmass where, except for spinel, the primary mineralogy has been replaced by carbonate and minor serpentine.

#### Ultramafic lamprophyres

**Fig. 3:**
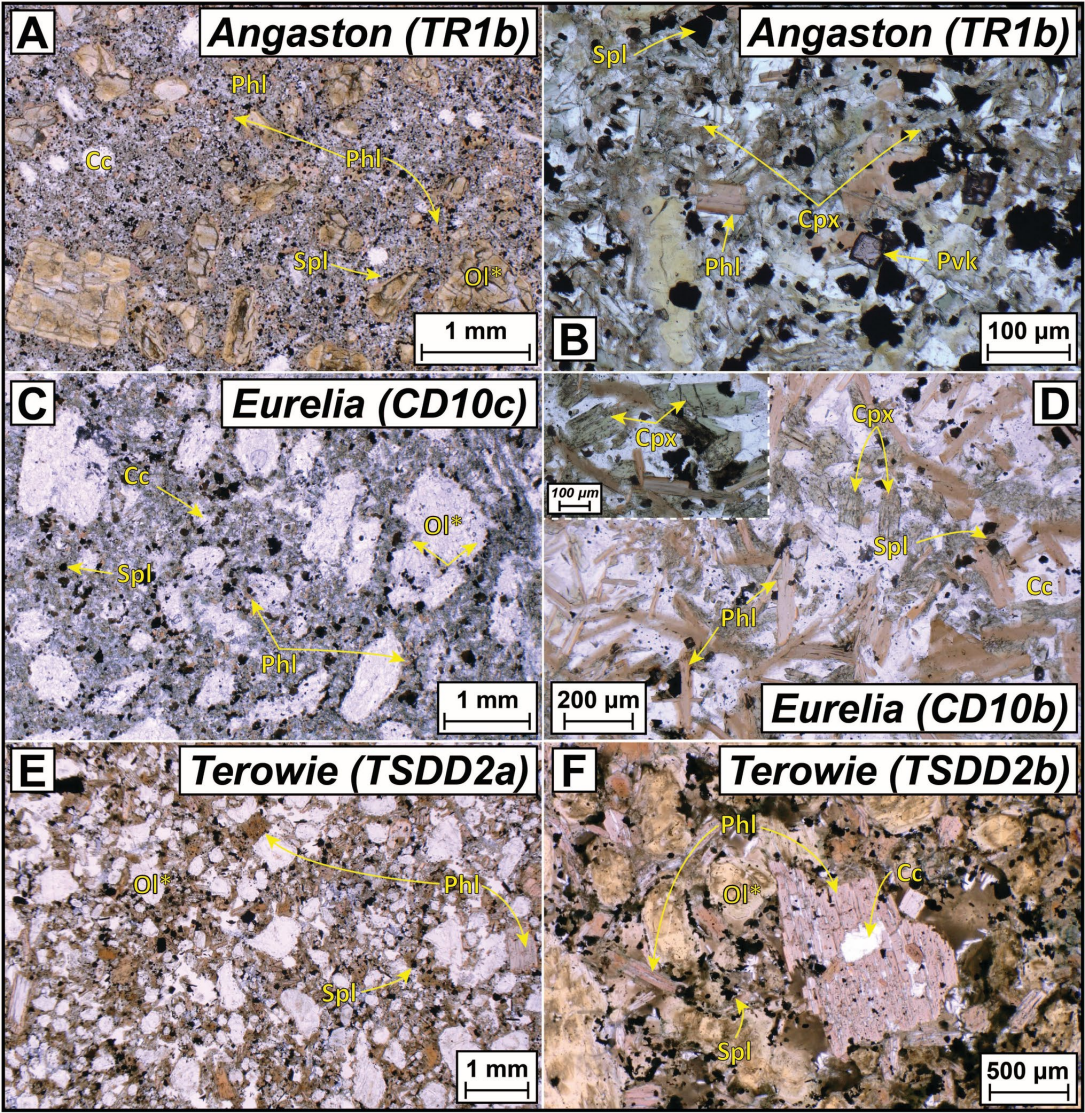
Representative plane-polarised transmitted-light photomicrographs for the three ultramafic lamprophyre localities sampled for this study: **A** and **B**) Angaston; **C** and **D**) Eurelia; **D** and **E**) Terowie. Abbreviations of mineral names: Phl – phlogopite; Spl – spinel; Pvk – perovskite; Ol* – pseudomorph after olivine; Cc – calcite; Cpx – clinopyroxene. See Table 1 for detailed descriptions

The Angaston (pipe Angaston-01) sample TR1b is micaceous with both phenocrysts and macrocrysts of phlogopite present (Fig. 3A,B). Relative to the localities described above, olivine macrocrysts and microcrysts are less prominent, but where present are replaced by serpentine. The groundmass is dominated by both phlogopite and clinopyroxene (Fig. 3B), which clearly rules out a kimberlite affinity for these samples. Perovskite and spinel are the other main phases while the mesostasis comprises carbonate and lesser amounts of serpentine. Samples TR1a and 1c represent more altered versions of TR1b where olivine is completely replaced by carbonate, fresh clinopyroxene is not observed and perovskite is also absent.

For Eurelia, sample CD10c contains olivine microcrysts replaced by carbonate, and often surrounded by perovskite and spinel. The groundmass also includes phlogopite and clinopyroxene with interstitial carbonate and serpentine (Fig. 3C). In contrast, sample CD10b is largely devoid of any pseudomorphs of olivine and is instead dominated by microphenocrysts of phlogopite and euhedral to subhedral microphenocrysts of clinopyroxene (~100–200 µm) with carbonate in the mesostasis (Fig. 3D). Sample CD10a is more highly altered with serpentinised olivine macrocrysts and chloritised phlogopite macro- and microcrysts with lesser spinel and perovskite. Altogether the Eurelia samples are also better classified as ultramafic lamprophyres (or perhaps olivine lamproites) rather than kimberlites.

Rocks from Terowie (locality Terowie 6) are micaceous, even more so than Angaston, with red–orange, zoned phlogopite macrocrysts and phenocrysts interspersed between olivine macrocrysts that are replaced by carbonate and/or serpentine (Fig. 3E,F). The phlogopite grains range from inclusion free laths to highly poikilitic crystals with inclusions of spinel, apatite, perovskite and earlier formed mica phenocrysts (Fig. 3F). Tetraferriphlogopite rims are also a feature of phlogopite grains in samples from Terowie. As with the samples from Angaston and Eurelia, clinopyroxene is also present within the groundmass but the grain size is considerably smaller. Spinel, serpentine and carbonate are the other main groundmass constituents. The classification of these rocks is discussed in more detail below.

### Whole-rock geochemistry

#### Major element geochemistry

**Fig. 4:**
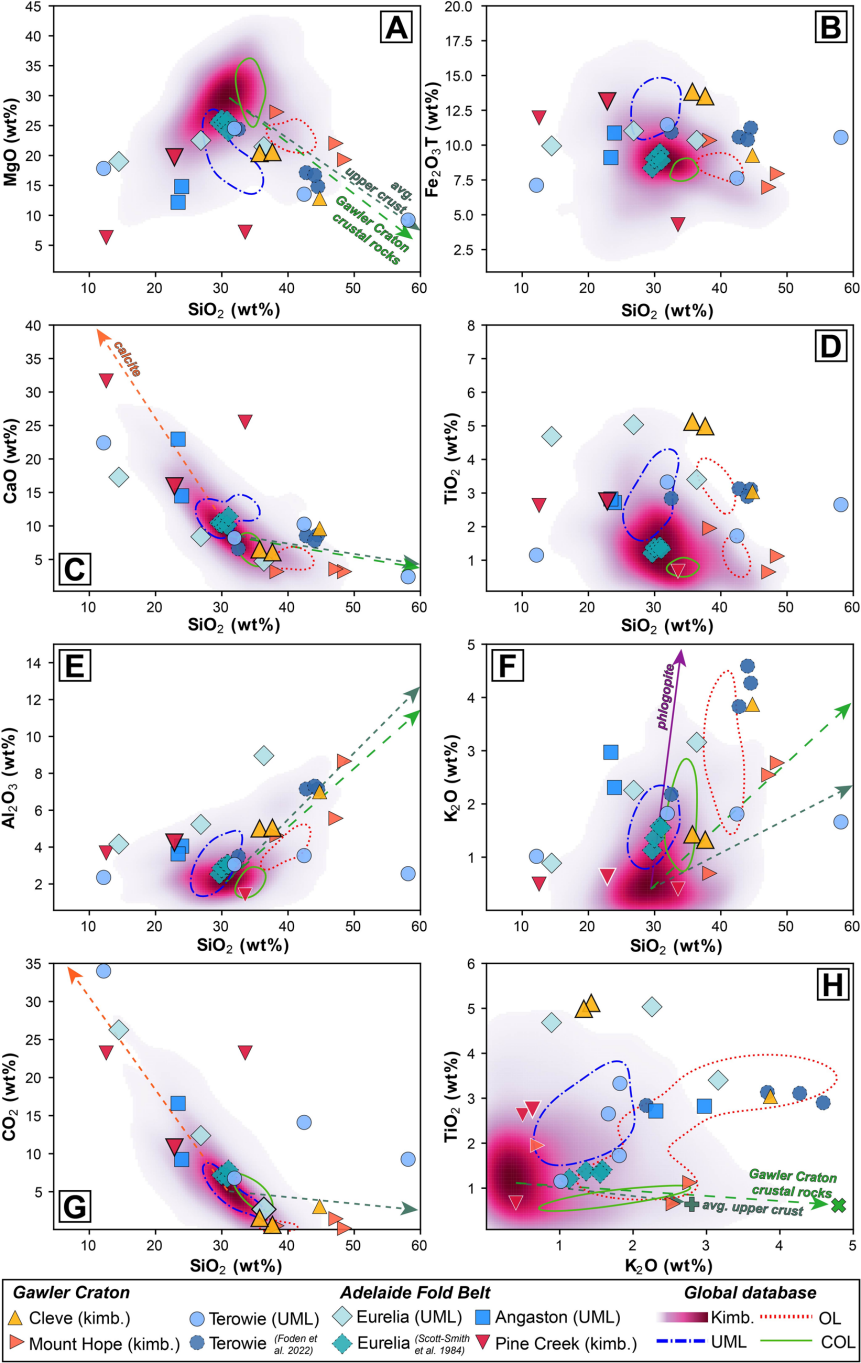
Major oxide co-variation charts for South Australian kimberlite and ultramafic lamprophyre (UML) samples: **A**) MgO; **B**) Fe_2_O_3_T (total Fe oxide, assuming all Fe to be ferric); **C**) CaO; **D**) TiO_2_; **E**) Al_2_O_3_; **F**) K_2_O; **G**) Na_2_O; **H**) CO_2_ versus SiO_2_, and **I**) TiO_2_ versus K_2_O. Coherent kimberlite samples are shown as larger symbols with thicker edges. These data are plotted against the global kimberlite database of Giuliani et al. (2025) using a gaussian kernel density estimation to highlight the most ‘typical’ compositions (constructed with pyrolite; Williams et al. 2020). Also plotted are compositional fields for UML, olivine lamproites (OL) and carbonate-rich olivine lamproites (COL). Each field represents the high-density area that denotes the 75 th percentile of values based on kernel density estimates for the data compiled. Also plotted are Terowie and Eurelia (Orroroo) data from Foden et al. (2002) and Scott-Smith et al. (1984), respectively. Vectors to calcite (Castillo-Oliver et al. 2018), phlogopite (Giuliani et al. 2016), and average upper continental crust (Rudnick and Gao 2014) and the average of typical Gawler Craton crustal rocks (Reid and Payne 2017) are also shown to demonstrate the control they may exert on the composition of these samples

A notable feature of these South Australian samples is the extensive major element variation, both within and between single localities. For example, SiO_2_ contents vary from 12.2 wt% to 58.1 wt%, TiO_2_ from 0.67 to 5.0 wt% and MgO from 6.3 to 27.3 wt% (Fig. 4; full data reported in Table S1). Regardless of the exact lithology, the samples show a moderately strong linear relationship between CaO and CO_2_ suggesting modal carbonate (calcite) exerts the predominant control and is evidenced by the vectors towards calcite compositions in Fig. 4C and G. Where samples deviate from this trend, it appears that interaction with upper crustal material may be the cause (green vectors in Fig. 4). Finally, Pine Creek, Terowie, Eurelia and Angaston all have samples with elevated CaO and CO_2_, consistent with the carbonate apparent in these rocks.

**Fig. 5:**
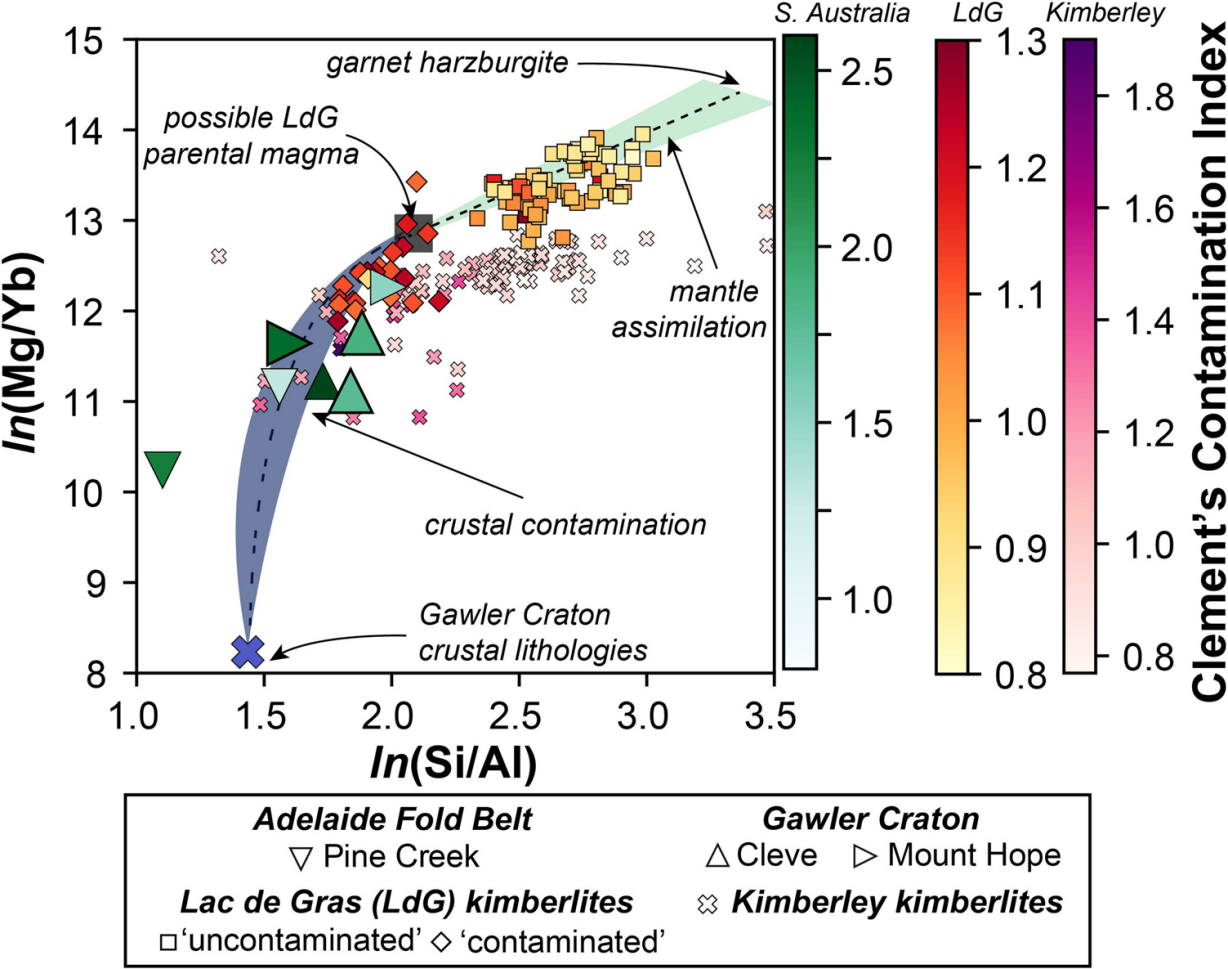
Assessment of crustal and mantle contamination for South Australian kimberlite samples using *ln*(Mg/Yb) versus *ln*(Si/Al) after Kjarsgaard et al. (2009; 2022). Coherent kimberlite samples are shown as larger symbols with thicker edges. Blue and green fields illustrate the impacts of crustal and mantle assimilation/contamination, respectively. Also plotted are bulk-rock data for Kimberley kimberlites (le Roex et al. 2003; Fitzpayne et al. 2021) and intentionally selected contaminated and uncontaminated Lac de Gras (LdG) kimberlites alongside the composition of a hypothetical LdG parental kimberlite melt (Kjarsgaard et al. 2009). Note all symbols have been coloured based on their Contamination Index (Clement 1982) value, with the related key on the right-hand side of the figure. The average of typical Gawler Craton crustal rocks is taken from Reid and Payne (2017). UML samples from this study are not plotted here as these contamination indices were designed exclusively for kimberlites

For the localities described above as kimberlites (Cleve, Mount Hope, Pine Creek) it is evident that their major element compositions both overlap with, and extend beyond, the ‘typical’ compositions of global hypabyssal kimberlites (Fig. 4). However, it is important to note that the three ‘kimberlite’ samples with elevated SiO_2_ contents (> 45 wt%) from both Cleve and Mt Hope, are all volcaniclastic and contain visible lithic fragments in thin section (Fig. 2A,D) and therefore it is likely that these ‘extreme’ compositions result from incorporation of fragmented crustal materials. This is consistent with these same samples also having Al_2_O_3_ contents > 5.5 wt% and an apparent trend towards crustal compositions in Fig. 4E. When using established indices for assessing the extent to which kimberlite whole-rock compositions have been impacted by crustal contamination (e.g., Clement 1982; Kjarsgaard et al. 2009) it is further apparent that the major element composition of these samples has been affected by crustal contamination (i.e., low *ln*(Si/Al) and *ln*(Mg/Yb); Clement’s Contamination Index values > 1.5; Fig. 5).

The samples classified above as UMLs (Terowie, Angaston and Eurelia), including those from Scott-Smith et al. (1984) and Foden et al. (2002) for the same/similar localities, tend to have elevated TiO_2_, K_2_O, Al_2_O_3_ and Fe_2_O_3_, consistent with the abundance of phlogopite and clinopyroxene. Relative to kimberlites, enrichments in TiO_2_, K_2_O, Al_2_O_3_ are also typical of UMLs and olivine lamproites globally (Fig. 4). Low MgO contents compared to typical kimberlites (and UML) are consistent with the common replacement of olivine by serpentine and carbonate.

#### Trace element geochemistry

**Fig. 6:**
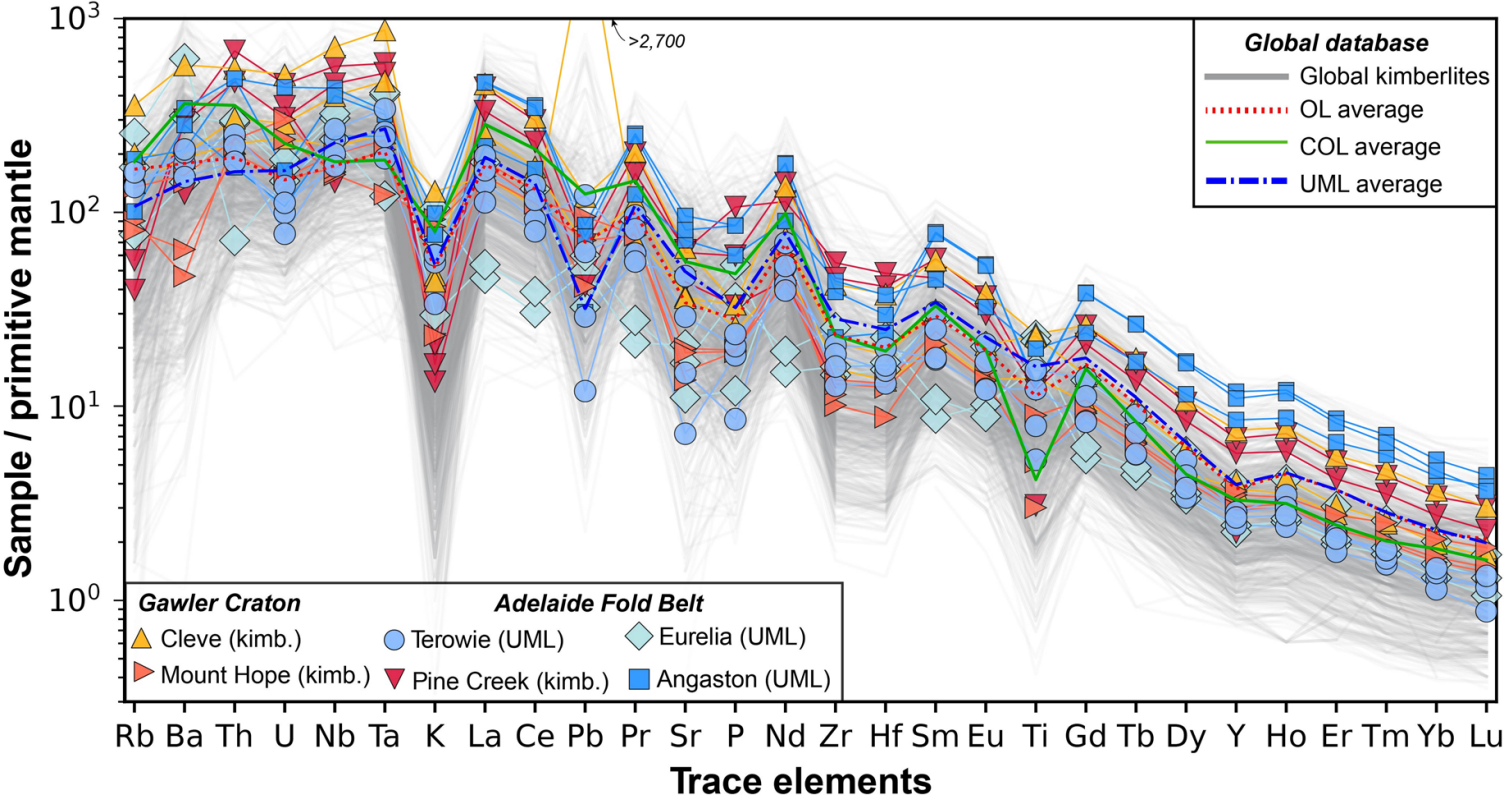
Primitive mantle-normalised bulk-rock trace element patterns for South Australian kimberlite and ultramafic lamprophyre (UML) samples plotted relative to global kimberlite compositions (grey lines) and average compositions for olivine lamproites (OL), carbonate-rich olivine lamproites (COL) and UMLs from Giuliani et al. (2025). Primitive mantle values from Sun and McDonough (1989)

The trace element contents of all samples in this study are characterised by a strong enrichment in incompatible elements, typical of kimberlites, UMLs, and olivine lamproites (Fig. 6). With few exceptions, the general patterns on a primitive-mantle normalised diagram (Fig. 6) are broadly similar including strong fractionation in the rare earth elements (La/Yb = 49—240) and negative anomalies for K, Pb, Sr-P, Zr-Hf and Ti. The key difference appears to be the magnitude of the observed anomalies, although in some instances the ‘direction’ of these anomalies can be reversed – such as the positive Pb anomalies for all Eurelia and certain Terowie and Cleve samples, as well as negative Nd anomalies for samples from Eurelia.

**Fig. 7:**
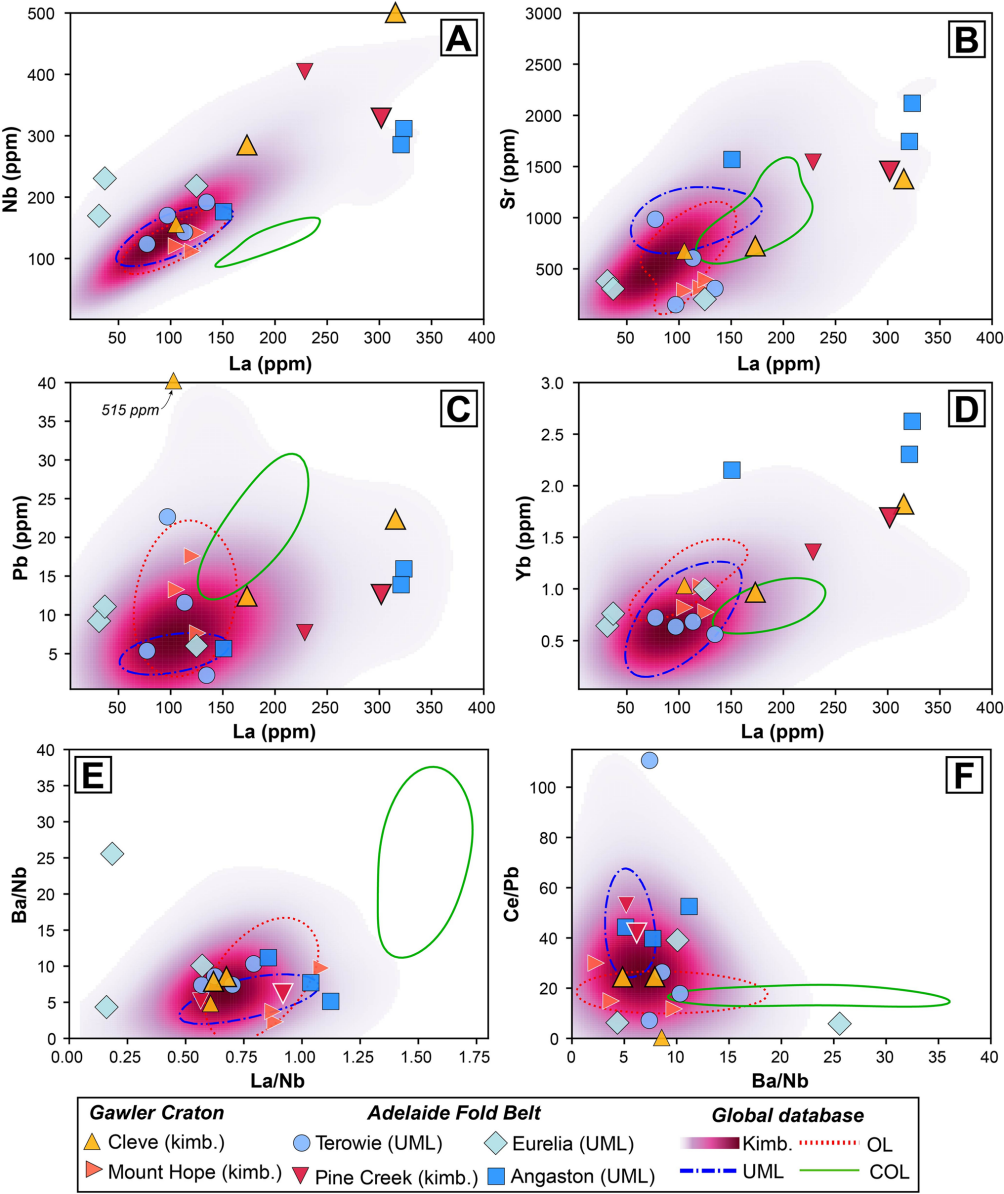
Incompatible trace element (A-D) and incompatible element ratio co-variation charts for South Australian kimberlite and ultramafic lamprophyre (UML) samples: **A**) Nb; **B**) Sr; **C**) Pb; **D**) Yb versus La; **E**) Ba/Nb versus La/Nb; and F9 Ce/Pb versus Ba/Nb. Coherent kimberlite samples are shown as larger symbols with thicker edges. These data are plotted against the global kimberlite database of Giuliani et al. (2025) using a gaussian kernel density estimation to highlight the most ‘typical’ compositions (constructed with pyrolite; Williams et al. 2020). Also plotted are compositional fields for UML, olivine lamproites (OL) and carbonate-rich olivine lamproites (COL). Each field represents the high-density area that denotes the 75 th percentile of values based on kernel density estimates for the data compiled

These differences in magnitudes and directions of anomalies are made clearer when inspecting the absolute concentration of trace elements, which, similar to major elements, vary widely within (e.g., La – Eurelia: 31–125 ppm), and between localities (e.g., Nb – Mount Hope 112 ppm versus Cleve 509 ppm; Fig. 7). The elevated levels of incompatible elements (e.g., La, Yb, Sr), including high-field strength elements (e.g., Nb), in samples from Cleve, Pine Creek and Angaston are consistent with these rocks having greater abundances of perovskite and oxide (spinel ± ilmenite) phases. The Pb content for the volcaniclastic Cleve sample CL-6a (> 500 ppm) appears anomalous and likely is the result of crustal contamination (see also le Roex et al. 2003). Excepting some of these more extreme concentrations, the absolute abundances of trace elements are within the range of those observed for kimberlites and UMLs found elsewhere.

Incompatible element ratios can also be used to distinguish between kimberlites and related rocks and provide insights into their potential source regions (e.g., le Roex 1986; 2003). When assessing the South Australian samples in this context, there is a substantial overlap with the compositions of global kimberlites, UMLs, as well as olivine lamproites, though they are readily distinguished from the carbonate-rich end member of olivine lamproites (Fig. 7). Some exceptions include two samples (CD10b, c) from Eurelia with low La/Nb, and one Terowie sample (TSDD2b) with elevated Ce/Pb, relative to those in kimberlites and UMLs.

### Radiogenic isotope geochemistry

#### Whole-rock, carbonate and perovskite Sr isotope compositions

**Fig. 8:**
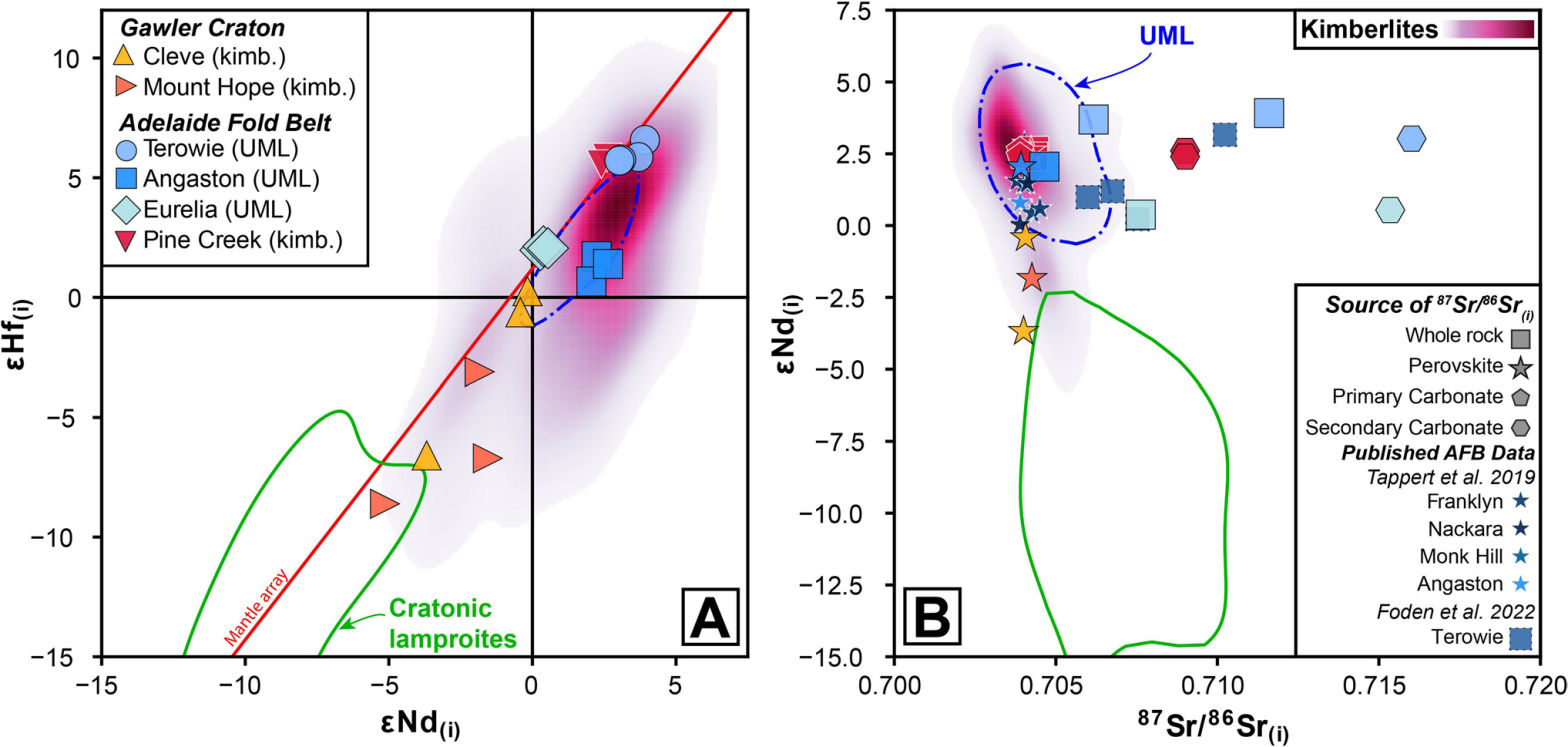
Radiogenic isotope co-variation charts for South Australian kimberlite and ultramafic lamprophyre (UML) samples: **A**) εHf_(i)_ versus εNd_(i)_ and **B**) εNd_(i)_ versus ^87^Sr^86^Sr_(i)_. In addition, whole-rock and perovskite-derived ^87^Sr^86^Sr_(i)_ values from Tappert et al. (2019) and Foden et al. (2002) for localities in the Adelaide Fold Belt (AFB) are also shown. Note that in panel B, different symbols are used to denote the ‘source’ of the ^87^Sr^86^Sr_(i)_ composition (i.e., whole-rock, perovskite, primary and secondary carbonate). These data are plotted against the global kimberlite database of Giuliani et al. (2025) using a gaussian kernel density estimation to highlight the most ‘typical’ compositions (constructed with pyrolite; Williams et al. 2020). Also shown is a field for UMLs and cratonic olivine lamproites (including carbonate-rich varieties) which represents the high-density area that denotes the 50.^th^ percentile of values based on kernel density estimates from the Giuliani et al. (2025) and Sarkar et al. (2025a) databases, respectively. Terrestrial mantle array is from Vervoort et al. (2011)

**Fig. 9:**
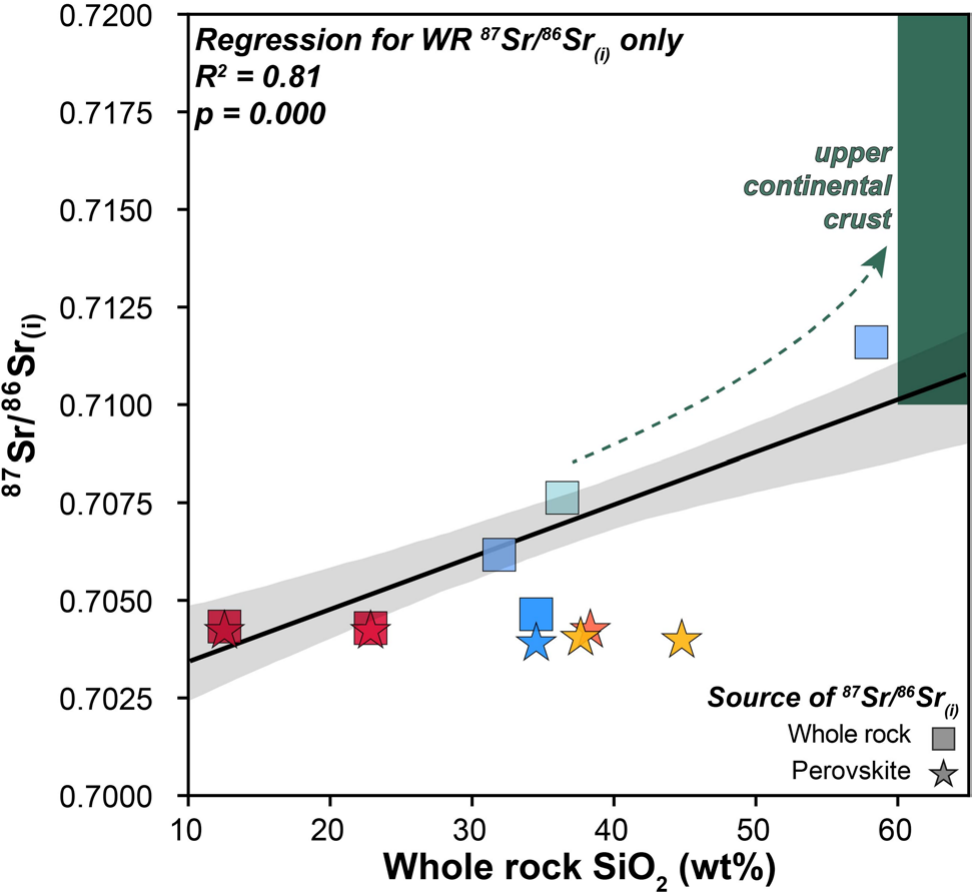
^87^Sr^86^Sr_(i)_ versus SiO_2_ covariation chart with a regression shown for whole-rock ^87^Sr^86^Sr_(i)_ against SiO_2_ content. Perovskite ^87^Sr^86^Sr_(i)_ are plotted against the whole-rock SiO_2_ for their respective sample. Also shown is a partial field for the upper continental crust where indicative SiO_2_ content is from (Rudnick and Gao 2014) and ^87^Sr^86^Sr_(i)_ ratios are taken from recent compilations by (Peucker-Ehrenbrink and Fiske 2019) and (Desem et al. 2025); note that the crust extends to more radiogenic ^87^Sr^86^Sr_(i)_ values than is shown here. *R*^*2*^ is the correlation coefficient, and the shaded field represents the 2 standard deviation uncertainty envelope for the regression line. Colours of symbols as per panels above

Both kimberlite and UML samples occupy a relatively narrow range in ^87^Sr/^86^Sr_(i)_ space (~ 0.704) based on in situ perovskite data (Fig. 8). Specifically, the ^87^Sr/^86^Sr weighted means of perovskites from two Cleve samples are 0.70406 ± 0.00010 (2SE, n = 13/18, sample CL6c; see methods for data filtering criteria and Table S3 for full dataset) and 0.70400 ± 0.00010 (n = 17/25; sample CL6a) with the following ^87^Sr/^86^Sr for perovskites from Angaston-01 (sample DD92 TR1b), Mt Hope-5 and Pine Creek-04, respectively: 0.70392 ± 0.00031 (n = 7/9), 0.70426 ± 0.00013 (n = 13/23), and 0.70424 ± 0.00012 (n = 19/23). These values are consistent with published perovskite values (0.70379–0.70524) from Tappert et al. (2019) for additional Terowie localities emplaced within the AFB (Franklyn, Nackara, Monk Hill) as well as Angaston (Fig. 8B). However, with the exception of Angaston (whole-rock ^87^Sr/^86^Sr_(i)_ = 0.70464) and Pine Creek samples (0.70430), these values diverge from whole-rock analyses (^87^Sr/^86^Sr_(i)_ > 0.710), which exhibit a strong positive correlation with bulk-rock SiO_2_ contents (R^2^ = 0.81; *p* = 0.000; Fig. 9).

The in situ carbonate analyses produce highly variable ^87^Sr/^86^Sr_(i)_ values, ranging from ~ 0.704 to ~ 0.716. In the case of the Pine Creek carbonatite sample BDDH1a, the ^87^Sr/^86^Sr_(i)_ composition of the carbonates (0.7039 ± 0.0002, n = 6), is indistinguishable to that of perovskite in the kimberlite sample BDDH1b (Table S4 for full dataset). Conversely, all the carbonates in Eurelia sample CD10c yield highly radiogenic values (0.71535 ± 0.0001; n = 13/21; 8 datapoints removed based on ^84^Sr/^86^Sr and/or ^87^Rb^86^Sr screening criteria; see methods). Carbonates in Terowie sample TSDDB3b are also predominantly very radiogenic Sr (0.71601 ± 0.0002; n = 19/28; 9 datapoints removed based on being statistical outliers; ~ 0.704–0.709). However, at least one analysis shows unradiogenic Sr (0.70399; not plotted) similar to perovskite compositions elsewhere in the AFB, and several carbonates have intermediate compositions between these radiogenic and unradiogenic endmembers, respectively.

#### Whole-rock Nd-Hf isotope compositions

Kimberlite and UML samples from the AFB generally display tight groupings in Nd-Hf space for each locality but show variations between different clusters (Fig. 8A). The Terowie UML and Pine Creek kimberlite samples have quite similar compositions at εNd_(i)_ +2.2 to +3.9 and εHf_(i)_ +5.7 to +6.6, indicative of sources characterised by long-term depletion of incompatible elements, whereas the Eurelia and Angaston UML samples plot towards more enriched (less radiogenic) compositions with εNd_(i)_ values ranging from +0.3 to +2.1 and εHf_(i)_ values from +0.7 to +2.1. It is noteworthy that the Angaston samples plot to the right of the Nd-Hf terrestrial array (Vervoort et al. 2011) and have ΔεHf_(i)_ of −3.7 to −4.6. The perovskite-derived εNd_(i)_ compositions of additional AFB localities from Tappert et al. (2019) broadly overlap with the values obtained in this study (εNd_(i)_ +0.0 to +3.1).

The Cleve and Mount Hope kimberlite samples from the Gawler Craton, in contrast, plot almost exclusively in the isotopically ‘enriched’ quadrant of Fig. 8 and show inter-sample heterogeneity. The two hypabyssal samples from Cleve (CL6b and CL6c) have restricted εNd_(i)_ compositions of −0.2 to −0.4 with εHf_(i)_ of +0.3 and −0.6, while the crustally contaminated, volcaniclastic sample (CL6a) plots towards more extreme compositions at εNd_(i)_ = −3.7 and εHf_(i)_ −6.6. The three Mount Hope samples exhibit greater scatter and extend to even more geochemically enriched compositions with εNd_(i)_ signatures ranging from −1.5 to −5.1 and εHf_(i)_ from −2.7 to −8.6. Such strongly negative εNd_(i)_ and εHf_(i)_ values are seldom observed in kimberlites (e.g., Alto Paranaiba; Woodhead et al. 2019) and are more typical of olivine lamproites (e.g., Smith 1983; Fraser et al. 1985; Nowell 2004) and some UMLs (e.g., Torngat, Tappe et al. 2008; Finland, Dalton et al. 2022).

## Discussion

### (Re)-Classification of the South Australian ‘kimberlites’

**Fig. 10:**
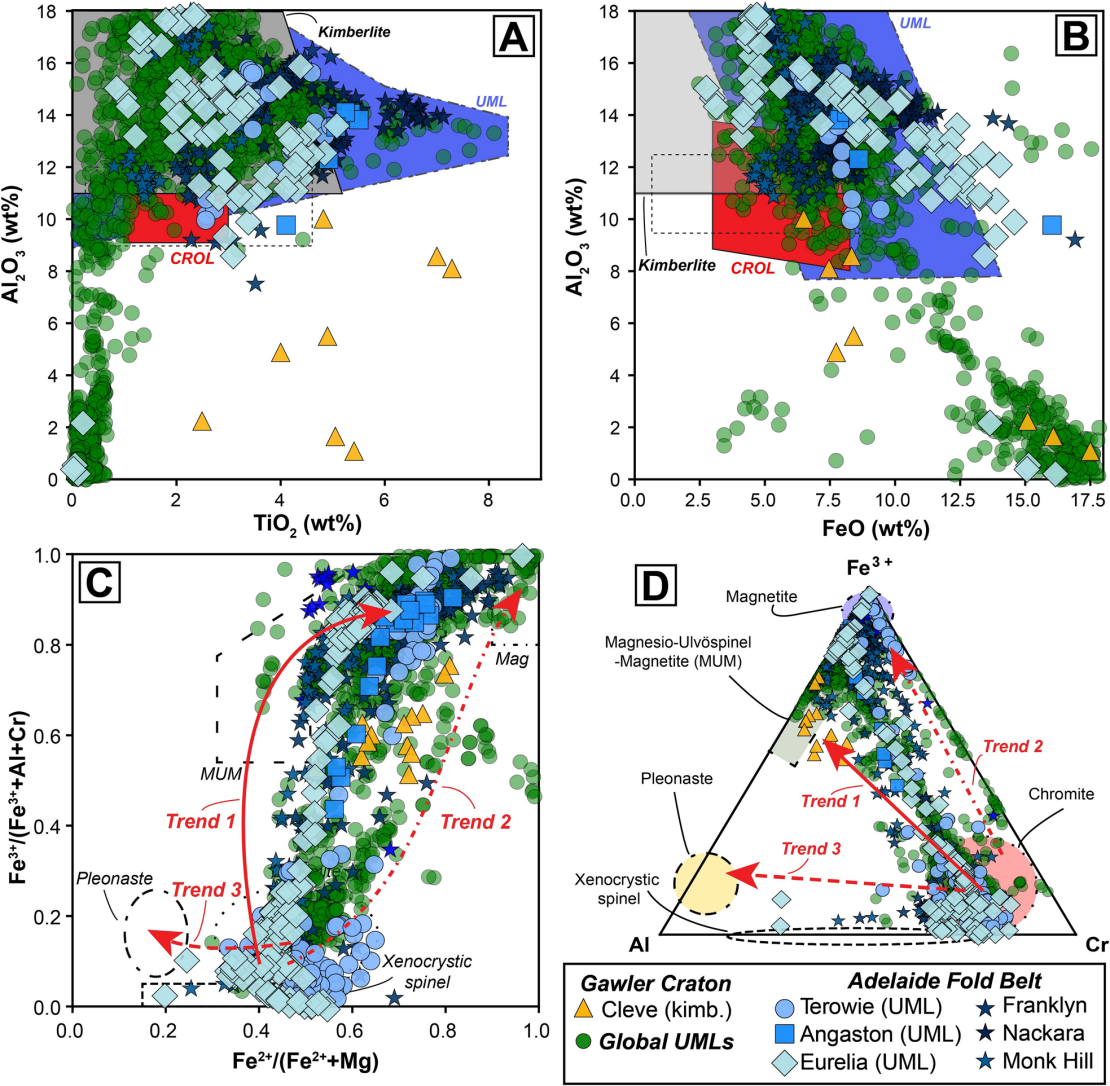
Mica (A and B) and Spinel (C and D) major element co-variation and ternary charts for South Australian kimberlites and ultramafic lamprophyres: **A**) Al_2_O_3_ versus TiO_2_ and **B**) Al_2_O_3_ versus FeO for mica; **C**) Fe^3+^/(Fe^3+^  + Al + Cr) versus Fe^2+^/(Fe^2+^  + Mg) and **D**) Fe.^3+^-Al-Cr ternary diagram for spinel. Also plotted is a new global compilation of > 1,500 mica and spinel analyses from ultramafic lamprophyres (Data from: Dalton et al. 2019; Nielsen et al. 2009; Sudholz et al. 2023; Tappe et al. 2006, 2004, 2009; Zozulya et al. 2020). These compiled data are available the supplementary Tables (ESM). Discrimination fields in A) and B) are updated from Kjarsgaard et al. (2022). Discrimination fields and magmatic evolution trends (red arrows) in C) and D) from Mitchell (1986) and Roeder and Schulze (2008)

As noted above, all the ultramafic bodies in the Gawler craton and AFB were initially all classified as kimberlites (Scott-Smith et al. 1984; Wyatt et al. 1994; Tappert et al. 2019). However, the micaceous nature, including abundant tetraferriphlogopite, and presence of clinopyroxene in the groundmass of samples from Angaston, Eurelia and Terowie (Fig. 3) is at odds with their classification as kimberlites and more consistent with ultramafic lamprophyres (and olivine lamproites; Tappe et al. 2005; Mitchell et al. 2019). In suggesting this reclassification, we acknowledge that tetraferriphlogopite rims can be observed in kimberlites (e.g., Dongre and Tappe 2019; Dalton et al. 2020; Viljoen et al. 2022) and highly micaceous kimberlites have been described elsewhere (e.g., West Africa; Taylor et al. 1994; Fitzpayne et al. 2023). However, the mica and spinel compositions published by Scott-Smith et al. (1984) and Tappert et al. (2019) for these and other Adelaide Fold Belt (AFB) occurrences also supports their reclassification based on their comparison with a newly created database of > 1,500 spinel and phlogopite analyses from worldwide UMLs (Fig. 10; supplementary Tables). TiO_2_ contents for phlogopite from Angaston and Terowie are all > 3 wt% while mica from Eurelia also shows Ti-enrichment up to 5 wt% (Tappert et al. 2019). Although such Ti enrichment is observed in some kimberlitic mica, it is a feature more typical of ultramafic lamprophyres (Tappe et al. 2004, 2006; Nielsen et al. 2009; Dalton et al. 2019). The FeO contents of phlogopite from Angaston, Terowie and Eurelia are possibly even more diagnostic, exhibiting strong overlap with other UML compositions and extending up to 14 wt%, far beyond typical kimberlite compositions, without a substantial tetraferriphlogopite component (Fig. 10B). Spinel compositions (Fig. 10C,D) provide a similar line of evidence, with matching compositions to global UMLs and predominantly plotting along a field which is intermediate between Trend 1 (typical of kimberlites) and Trend 2 (typical of olivine lamproites). In addition, the bulk K_2_O contents for the UML samples are on average much higher than those observed for the freshest, coherent kimberlite samples in this study as well as typical kimberlite compositions elsewhere (Fig. 4). Finally, while the presence of mica and clinopyroxene alone does not preclude a petrographic classification as an olivine lamproite, the average Nd-Hf isotopic composition of samples from Angaston (εNd_(i)_ + 2.3; εHf_(i)_ = + 1.3), Eurelia (εNd_(i)_ + 0.4; εHf_(i)_ = + 2.1), and Terowie (εNd_(i)_ + 3.4; εHf_(i)_ = + 6.0) do not exhibit the levels of geochemical enrichment typical of cratonic lamproites globally (Fig. 8; Sarkar et al. 2025a, b) and are again, more typical of ultramafic lamprophyres (Fig. 8; Giuliani et al. 2025).

### Influence of crustal contamination

Prior to making petrogenetic inferences based on the new geochemical data presented here, it is critical to appraise the role of crustal contamination in causing any of the observed geochemical variability. For example, when considering the three UML localities discussed above (Terowie, Angaston, and Eurelia), it is striking that there is minimal, if any, inter-sample variation in their respective εNd_(i)_ and εHf_(i)_ compositions, despite their whole-rock and carbonate ^87^Sr/^86^Sr_(i)_ signatures varying significantly (~ 0.704 to ~ 0.716; Fig. 8). In contrast, the ^87^Sr/^86^Sr_(i)_ values obtained via analysis of perovskite for any of the South Australian localities (kimberlite or UML) exhibit a small range and are less radiogenic than all whole-rock and the majority of the carbonate analyses. This discrepancy between perovskite and bulk-rock Sr isotopes is a common observation in kimberlites and related rocks elsewhere (e.g., Paton et al. 2007a; Woodhead et al. 2009) and is generally attributed to a combination of physical entrainment of crustal lithics and hydrothermal alteration (Giuliani et al. 2025). In this case the direct correlation between whole-rock ^87^Sr/^86^Sr_(i)_ and SiO_2_ contents for all the AFB samples (UML and kimberlites – whole rock Sr isotopes not measured in the Gawler kimberlites) (Fig. 9) supports the addition of crustal fragments as the primary control on these Sr isotope variations.

Regarding the large range in Sr isotopic signature of the carbonates from the AFB localities (0.704 to 0.716), it should be acknowledged that previous studies have demonstrated that multiple generations and/or textural types of carbonates in the same kimberlite can exhibit similar or even greater variance in ^87^Sr/^86^Sr_(i)_ values (e.g., Exley and Jones 1983; Giuliani et al. 2017; Castillo-Oliver et al. 2018, 2020). In the case of the Pine Creek carbonatite sample (BDDH1a; 0.7039 ± 0.0002) it is evident that the carbonate has faithfully recorded the magmatic signature, given its indistinguishable isotopic composition from perovskite in a kimberlitic sample from the same body (Pine Creek-04; BDDH1b; 0.70424 ± 0.00012; Fig. 8). This contrasts strongly with the carbonate ^87^Sr/^86^Sr_(i)_ systematics from Eurelia (0.71535 ± 0.0001) and Terowie (0.71601 ± 0.0002) where almost all analyses returned values that are far more radiogenic than typical of kimberlites or UMLs including analyses of perovskite (0.7038—0.7052; Tappert et al. 2019; this study) in the AFB (Fig. 8). These highly radiogenic values are likely to be indicative of carbonate precipitation from late-stage C-O–H fluids which are substantially influenced by the input of crustal-derived Sr. In the case of Terowie, it is interesting that at least one carbonate returned a likely magmatic signature (i.e., ~ 0.704) and six carbonates returned values (~ 0.706 to ~ 0.709; Table S4) that are intermediate between this signature and one more typical of crustal contamination. These data suggest that, although apparently very pervasive, these crust-derived (or crust-influenced) hydrothermal fluids have not completely replaced/modified all of the ‘primary’ magmatic carbonate in the Terowie samples. This observation underscores the importance of in situ analysis of carbonates in kimberlites and related rocks, particularly given these minerals are likely to be the main sink of Sr in ‘fresh’ kimberlites (e.g., Fitzpayne et al. 2023).

Importantly, for all samples from the AFB, there is no correlation between εNd_(i)_ and εHf_(i)_ with SiO_2_ compositions (nor any other indicators for crustal contamination; Fig. S2) and we therefore conclude that these signatures have not been influenced by syn- or post-emplacement crustal contamination. This interpretation is consistent with the limited εNd_(i)_ and εHf_(i)_ variability for this sample suite. In contrast, the kimberlite samples from the Gawler Craton exhibit greater inter-sample differences and, as noted above, have isotopic compositions that are far more geochemically enriched than would be expected for typical kimberlites (Fig. 8), all of which requires explanation.

**Fig. 11:**
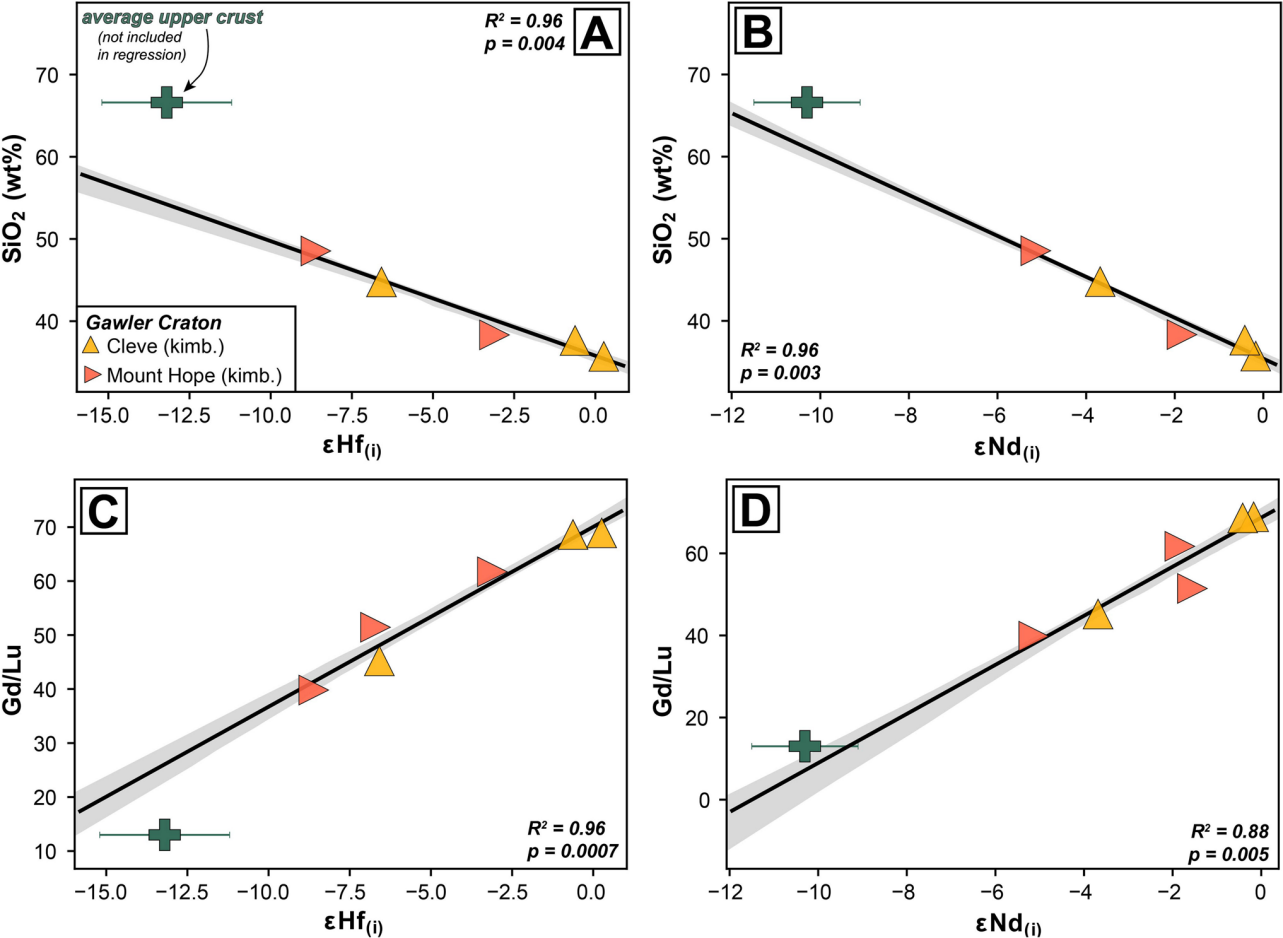
Co-variation charts of bulk-rock composition versus Nd-Hf isotopes for Gawler Craton kimberlite samples: **A**) SiO_2_ versus εHf_(i)_; **B**) SiO_2_ versus εNd_(i)_; **C**) Gd/Lu versus εHf_(i)_ and **D**) Gd/Lu versus εNd_(i)_. Also plotted for reference is the average composition of the upper continental crust with values taken from Rudnick and Gao (2014) and Chauvel et al. (2014). *R*^*2*^ is the correlation coefficient, and the shaded field represents the 2 standard deviation uncertainty envelope for the regression line

Unlike Sr, whole-rock Nd and Hf isotope compositions are typically thought to be representative of the ‘primary’ magmatic signature of kimberlites given their high concentrations relative to crustal rocks (see Rudnick and Gao 2014; Giuliani et al. 2025) and the fact these elements are typically fluid immobile. In order to assess the role of crustal contamination on the εNd_(i)_ and εHf_(i)_ compositions of the Gawler Craton kimberlites we have plotted these values against major element concentrations and trace element ratios (Fig. 11) which are considered to be sensitive to crustal contamination (le Roex 2003; Kjarsgaard et al. 2009). There are striking, statistically significant correlations (*p* < 0.05) between εNd_(i)_ and εHf_(i)_ with SiO_2_ (R^2^ = 0.96 and 0.96, respectively), Al_2_O_3_ (R^2^ = 0.84 and 0.81; not shown), Gd/Lu (R^2^ = 0.88 and 0.96; selected as a proxy for MREE/HREE) and Nb/U (R^2^ = 0.93 and 0.80; not shown), among many others. Conversely, no such correlations are apparent for AFB samples (Fig. S2). The samples with the highest SiO_2_ and Al_2_O_3_ have the most negative (i.e., most geochemically enriched, less radiogenic) εNd_(i)_ and εHf_(i)_ values, as is the case for those with the lowest Nb/U and Gd/Lu. This is consistent with crustal contamination considering that the upper continental crust has an average composition of εNd_(i)_ −10.3 ± 1.2 and εHf_(i)_ −13.2 ± 2.0 (Chauvel et al. 2014) and, similarly, the host granites and granodiorites of the southern Gawler Craton crust range from −3 to −20 for εNd_(i)_ and −3 to −15 for εHf_(i)_ (Nebel et al. 2007; Fraser et al. 2010; Champion 2013; Reid and Payne 2017; Curtis and Thiel 2019). Furthermore, if the anomalous Pb value of Cleve sample CL-6a (515 ppm) is excluded, a very strong correlation also exists between εNd_(i)_ and εHf_(i)_ with Pb/Nb (R^2^ = 0.82 and 0.91; not shown) where Pb is well known to be a useful determinant of crustal contamination (le Roex 2003). From this evidence it is clear that the unusually enriched isotopic signatures of the volcaniclastic samples Cleve and Mount Hope samples are unlikely to reflect the primary isotopic signature of their mantle source regions and instead reflect significant isotopic perturbation. Conversely, the two hypabyssal kimberlite samples from Cleve (CL-6b, 6c; Table 1) exhibit SiO_2_ and Al_2_O_3_ contents and Nb/U and Gd/Lu ratios inconsistent with substantial crustal contamination, hence suggesting that their Nd-Hf isotopes are representative of their mantle-derived kimberlite melt compositions (Fig. 4, Fig. 8, Fig. 11). Finally, perovskite analyses from two Cleve and one Mount Hope sample yielded indistinguishable ^87^Sr/^86^Sr_(i)_values of ~ 0.7041 which is similar to the values of perovskite in the AFB samples and a testament to the robustness of perovskite as a recorder of primary magmatic signatures (e.g., Paton et al. 2007a; Woodhead et al. 2009).

### Spatiotemporal controls on magmatism on the South Australian Craton

#### Gawler Craton versus Adelaide Fold Belt

It is noteworthy that the AFB hosts kimberlitic and ultramafic lamprophyre magmatism whereas the Gawler Craton appears to exclusively host kimberlites. Regarding the coexistence of UMLs and kimberlites in the AFB, the question is whether there is a petrogenetic relationship between these types of magmatism. Not only do kimberlites and UMLs share similar incompatible trace element ratios and Sr–Nd-Hf isotope compositions globally (e.g., Figs. 7, 8; see also Pearson et al. 2019; Giuliani et al. 2025), they are also co-located on a number of cratons and mobile belts worldwide, for example in Russia (Kola, Chadobest and Ilbokich; Beard et al. 1996; Nosova et al. 2020), Finland (Kuusamo; Dalton et al. 2019, 2022), Greenland (Larsen and Rex 1992; Nielsen et al. 2009; Tappe et al. 2011), Canada (Torngat, Labrador; Tappe et al. 2008), and India (Narayanpet and Wajrakarur; Paton et al. 2009).

Along the Labrador strait, the variation in the type of ultramafic to alkaline magmatism over long periods of time (i.e., > 1500 Ma) has been proposed to be the result of changes in lithospheric thickness and therefore the depth and extent of melting (Larsen and Rex 1992; Tappe et al. 2007). However, when broadly coeval Neoproterozoic UMLs and kimberlites from this same region were investigated (i.e., Maniitsoq, Sarfartoq, Sisimut and Torngat) it was argued that there was no relationship between rock type and depth of melting (Nielsen et al. 2009; Sand et al. 2009) despite the Maniitsoq kimberlites occurring in the Archean core of the craton and all UMLs (aillikites) occurring in the Paleoproterozoic mobile belts. Instead, the unique characteristics of the UMLs, relative to kimberlites, is suggested to be the result of interaction with metasomatised subcontinental lithospheric mantle (SCLM) where metasomatism may be more prevalent at craton margins (e.g., Nielsen et al. 2009; Tappe et al. 2011). A similar scenario was envisaged by Dalton et al. (2019, 2022) for UML magmatism in Finland where it was modelled that an asthenospheric melt originating from a similar source to kimberlites (e.g., Giuliani et al. 2021) would gain its UML ‘characteristics’ via mixing with an enriched melt (~ 15–20 vol%) sourced in the metastomatised SCLM.

**Fig. 12:**
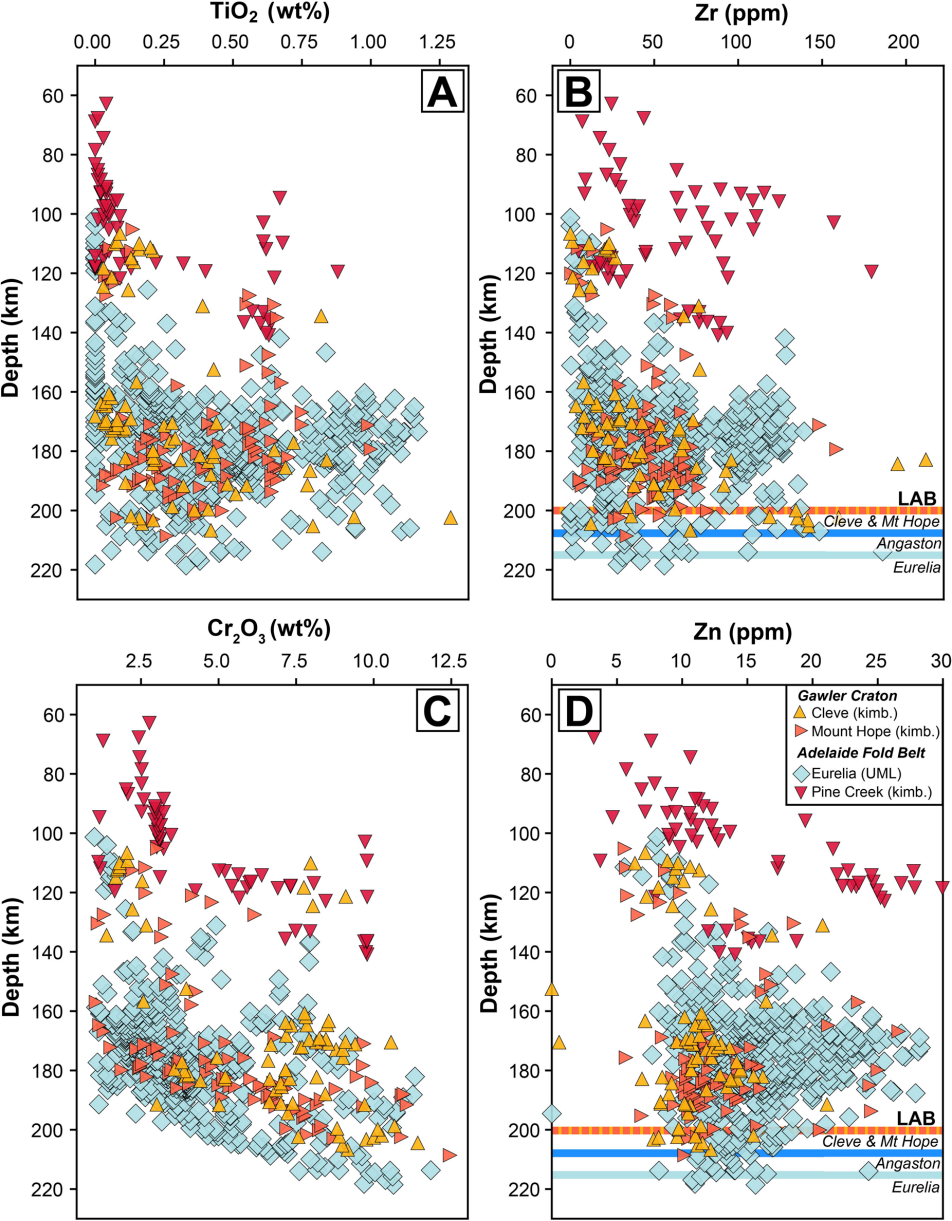
Co-variation charts of depth versus composition for pyrope garnet xenocrysts from South Australian kimberlites and ultramafic lamprophyres (UML). **A**) Depth versus TiO_2_; **B**) Depth versus Zr; **C**) Depth versus Cr_2_O_3_ and **D**) Depth versus Zn. All data and lithosphere-asthenosphere boundary depth estimates are from Sudholz et al. (2022). Garnet P–T was calculated using the Ni-in-garnet geothermometer of Sudholz et al. (2021b) and extrapolating onto the local geotherm defined by the clinopyroxene P–T (Nimis and Taylor 2000; Sudholz et al. 2021a). Note that garnet xenocryst data are not available for Angaston and clinopyroxene xenocryst data are not available for Pine Creek and hence there is no LAB estimate for this locality

Here we employ available garnet xenocryst and paleogeotherm data (Sudholz et al. 2022) to ascertain whether or not there are notable differences in the lithospheric structure and composition beneath the Mount Hope and Cleve kimberlites of the Gawler Craton and the Eurelia UML and Pine Creek kimberlite of the AFB (Fig. 12; note that garnet data are not available for Angaston). The Cleve and Mount Hope kimberlites clearly sampled similar lithospheric mantle material and have identical lithosphere-asthenosphere boundary (LAB) estimates at ~ 190 to 205 km. It is estimated that the LAB beneath the Angaston and Eurelia UMLs may be marginally deeper at ~ 205 to 215 km and ~ 210 to 220 km, although these paleogeotherms are based on just 7 and 5 clinopyroxene grains. In addition to limited mantle sampling, it is difficult to draw firm conclusions about differing LAB depths between these specific localities, given the inherent challenges associated with estimating lithospheric thickness due to uncertainties in P–T estimates of geothermobarometers combined with necessary assumptions of crustal thickness, crustal heat production and mantle potential temperature (e.g., Mather et al. 2011; Sarkar et al. 2025b). Although the exact depths of the LAB may be uncertain, the composition of Eurelia pyrope garnets (i.e., enrichments in TiO_2_, Zr and Zn at broadly similar Cr_2_O_3_ contents; Fig. 12) from the lower lithosphere suggests this region of the AFB has experienced more metasomatic enrichment relative to the SCLM beneath the Gawler. Furthermore, recent geophysical assessments of South Australia (e.g., Skirrow et al. 2018; Birkey et al. 2021; AusLAMP 2023) have detected clear differences (e.g., resistivity, shear-wave velocity) in lithospheric architecture between the Gawler Craton and the AFB. This variance has been attributed to LAB depth and lithological variation between these two regions, consistent with the findings of Sudholz et al. (2022) who noted a higher prevalence of metasomatic ‘pockets’ beneath the AFB based on the composition of garnet and clinopyroxene xenocrysts. Similar to the North Atlantic Craton example described above, while there is no clear relationship between lithospheric thickness and the type of magmatism expressed at the surface, it is possible that interaction between similar carbonate-rich precursor melts and deep SCLM that was more intensely metasomatised in the AFB may dictate why UMLs are absent from the Gawler Craton and more prominent in the AFB.

Previous work has suggested that the composition of olivine in kimberlites can be employed to trace the extent of lithospheric mantle interaction including the presence of metasomatised Fe-Ti–rich lithologies (e.g., Giuliani et al. 2020; Howarth et al. 2022; Viljoen et al. 2022). However, no olivine data is available for the examined samples. Alternatively, enrichment in phlogopite, locally combined with more radiogenic Sr isotopes, can also be considered a hallmark of contribution by metasomatised SCLM in asthenospheric melts (e.g., Sarkar et al. 2022, 2025a; Fitzpayne et al. 2023; Giuliani et al. 2025) because no potassic minerals with high K/Na such as mica and K-richterite are stable in the asthenosphere (Konzett 1997; Trønnes 2002). Comparison of bulk-sample K_2_O contents in the freshest samples from this study, including two coherent hypabyssal kimberlites from Cleve and one from Pine Creek (see larger symbols in Fig. 4), shows lower K_2_O and therefore mica contents in the kimberlites compared to the UMLs. This observation supports a role for more extensive interaction with mica-bearing lithospheric lithologies in the genesis of UMLs. On the other hand, the similar Sr isotope compositions of perovskite in kimberlites and UMLs in the southern Gawler Craton and AFB (Fig. 8) requires either less radiogenic asthenospheric sources for the kimberlites or, more likely, limited contribution by radiogenic Sr from lithospheric mica perhaps because of a young age of mantle metasomatism. The variable Nd-Hf isotope compositions of kimberlites and UML from each cluster lend support to some variability in the asthenospheric source – if Nd and Hf isotopes in kimberlites and UMLs are minimally modified by interaction with metasomatised SCLM. However, they do not support a more geochemically-depleted source (i.e. with less radiogenic Sr) for kimberlites because kimberlites from Cleve (average εNd_(i)_ −0.3; εHf_(i)_ −0.2) and Pine Creek (average εNd_(i)_ +2.5; εHf_(i)_ +5.7) plot at the opposite ends of the spectrum of Nd-Hf isotope compositions observed for the least crustally-contaminated samples in this study (Fig. 8, Fig. 11).

In conclusion, while it is difficult to disentangle the possible competing influence of source and SCLM compositions, we suggest that the more heavily metasomatised lithosphere beneath the AFB (as indicated by petrological and geophysical evidence) has locally dictated the major element composition (and therefore mineralogy) of ascending carbonate-rich melts, resulting in the genesis of UMLs. In addition, on the basis of isotopic data for the freshest available samples, we also tentatively speculate that asthenospheric source region heterogeneity exists between the AFB and Gawler Craton localities.

#### Temporal trend in the Adelaide Fold Belt

**Fig. 13:**
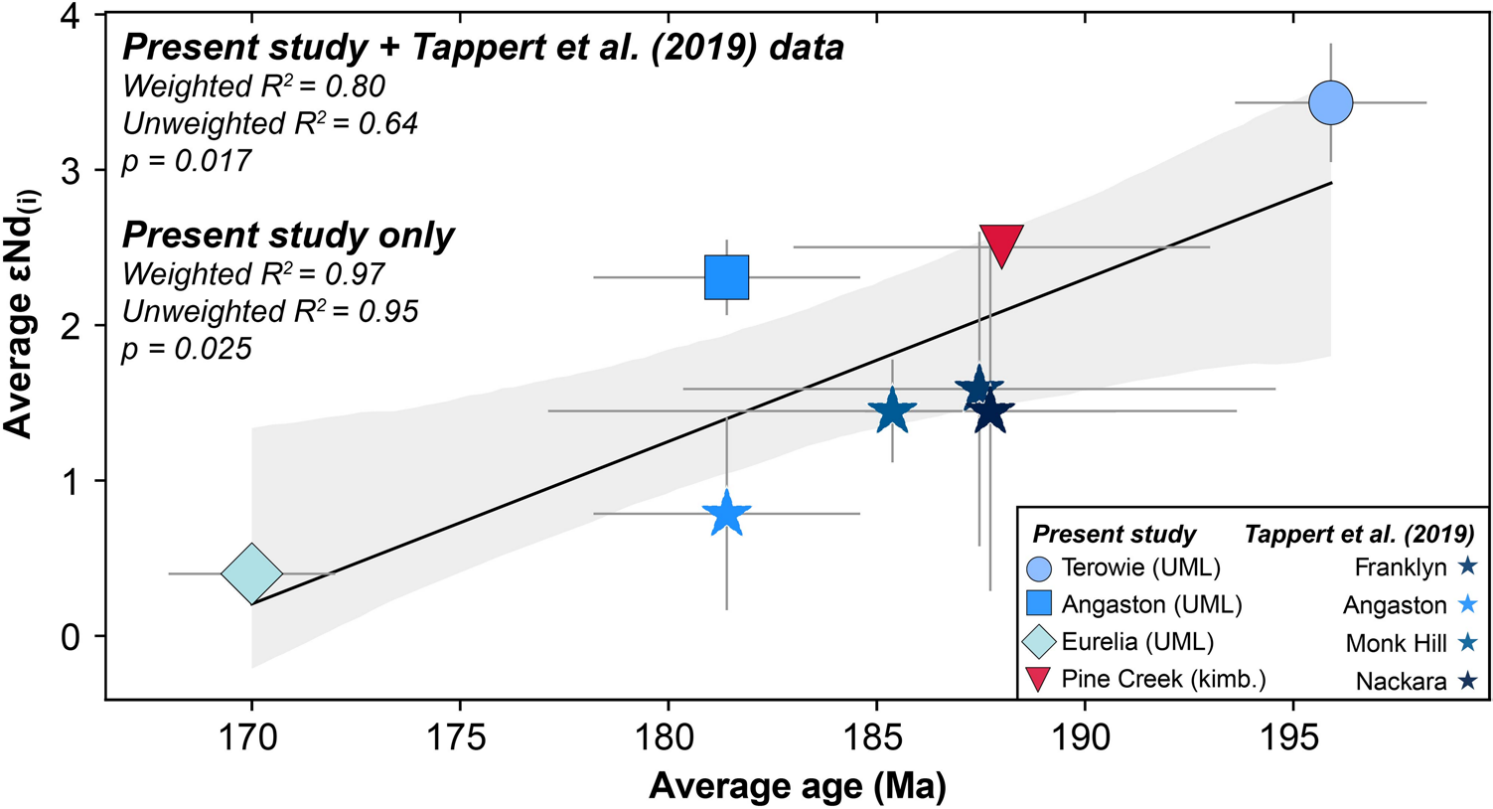
εNd_(i)_ isotope compositions versus emplacement age chart for kimberlite and ultramafic lamprophyre (UML) for localities from the Adelaide Fold Belt only. Also plotted are perovskite εNd_(i)_ values from Tappert et al. (2019). *R*^*2*^ is the correlation coefficient, where both an unweighted and weighted value, based on the uncertainty in individual data points, is shown. The shaded field represents the 2 standard deviation uncertainty envelope for the regression line

**Fig. 14:**
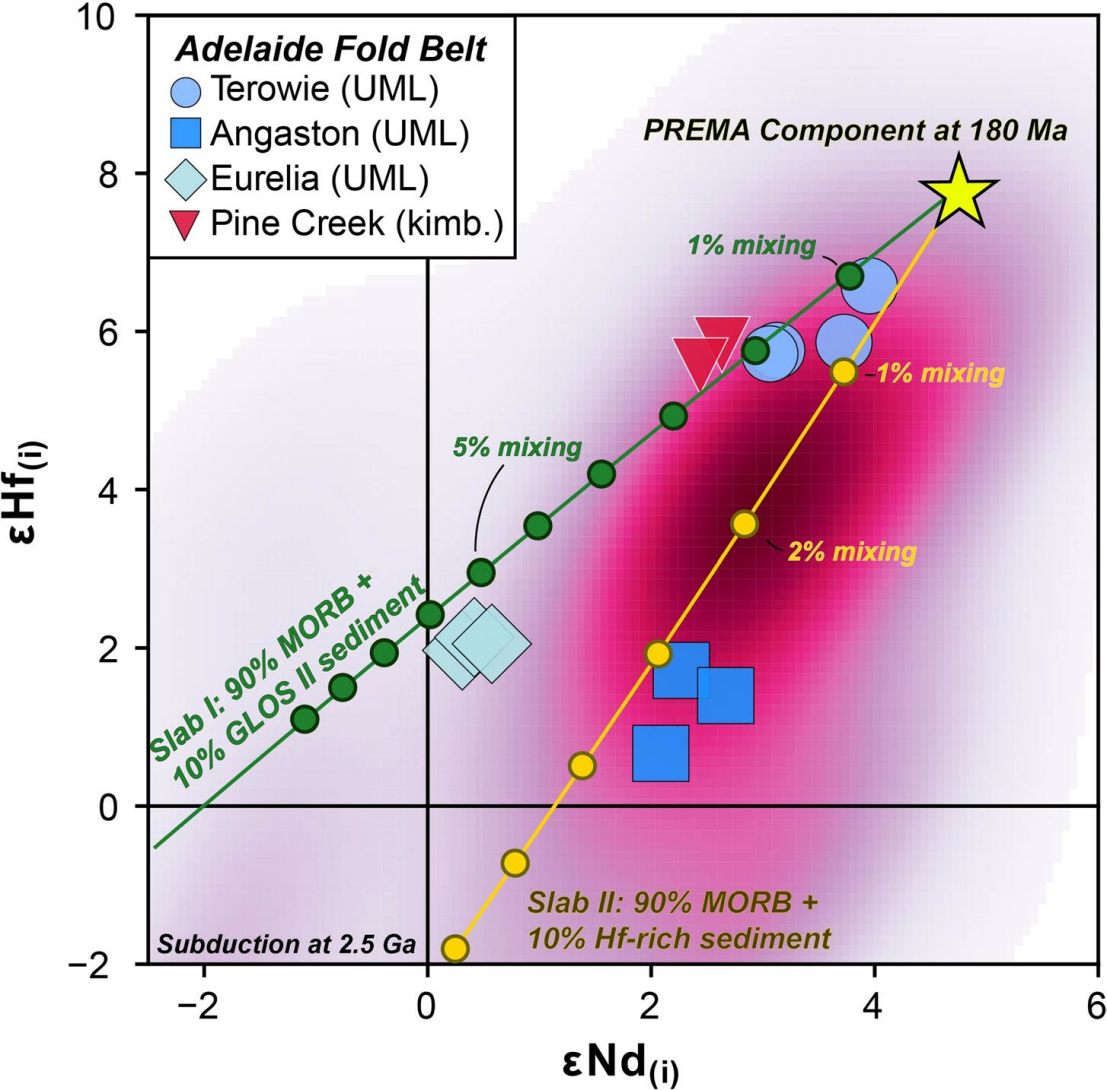
εHf_(i)_ versus εNd_(i)_ radiogenic isotope co-variation chart for Adelaide Fold Belt localities showing binary mixing relationship between a geochemically depleted asthenospheric PREMA component (Giuliani et al. 2021) and slab material subducted at 2.5 Ga. Subducted material is composed of 90% E-MORB (Gale et al. 2013) and 10% sediment, represented by either GLOSS II of Plank (2014) (Slab I) or the coarse Hf-rich sediment of Bayon et al. (2009) (Slab II). Compositions of subducted components adjusted to account for subduction-induced, sediment-fluid modification following Stracke et al. (2003). See Table S6 for details of model parameters

While it is not possible to identify a spatial control on the geochemical variation in the AFB samples, we have identified a possible temporal relationship between emplacement age and Nd isotope composition (Fig. 13). Note that Hf isotope compositions also appear to vary with time but there are fewer data and therefore the relationship is considered less robust (Fig. SS3). Conversely, Sr isotopes in perovskite are largely invariant and do not show any obvious temporal trend (not shown). We observe that the younger intrusions display increasingly enriched isotopic compositions, progressing from around εNd_(i)_ +4 to +0.5 (εHf_(i)_ +7 to +1) across the ~ 25 million years of kimberlite and UML emplacement recorded by these samples (Fig. 13; Fig. S3). This observation is inconsistent with the hypothesis that progressive melt extraction from a given mantle source would result in the continual geochemical depletion of that source (as observed in the Lac de Gras kimberlites; Tovey et al. 2021). One possible explanation is that the variation in the UML chemistry in particular is driven by varying degrees of interaction between the asthenospheric precursor melt and an extremely heterogeneous SCLM. However, this would suggest that the observed temporal pattern (Fig. 13) is entirely coincidental. Alternatively, a mechanism for progressive geochemical enrichment of the convecting mantle source beneath the AFB, which we suggest is common to all Jurassic AFB eruptions, could be invoked. Indeed, a similar temporal pattern was observed for the Neoproterozoic Kaavi-Kuopio kimberlites in Finland, for which it was suggested that progressive incorporation of subducted material into the kimberlite source region could successfully reconcile the observed isotopic compositions (Dalton et al. 2022). Mixing subducted slab material composed of both MORB and sediment with a convecting mantle source component equivalent to the depleted kimberlite PREMA (PREvalent MAntle; Zindler and Hart 1986) source region (Giuliani et al. 2021) shows that just minor contributions of slab material (~3 to 6%) can account for the shift in Nd-Hf isotopic compositions from Terowie through to Eurelia (Fig. 14) while also closely matching the data in Sr–Nd space (Fig. S4). Full modelling parameters are provided in Table S6 (see also: Dalton et al. 2022; Sarkar et al. 2023). The mixing proportions required depend on the composition chosen for each slab endmember and the age of subduction of the slab. Here (Fig. 14) we have selected a slab component comprising 90% MORB and a 10% sediment package akin to GLOSS II of Plank (2014) or an Hf-rich sediment from Bayon et al. (2009). Any composition between the subducted endmembers employed in this model would satisfy the observed compositions. Decreasing the age of subduction from 2.5 Ga to 1.5 Ga, for example, increases the volume required to a still reasonable ~10–12%. We therefore suggest that the progressive incorporation of subducted crustal material is a plausible explanation for the observed enrichment in the younger intrusions of the AFB.

This interpretation leads us back to the question posed in the Introduction as to the role of subducted material along the southern palaeo-margin of Pangea in the genesis of South Australian Jurassic kimberlites and UMLs – as previously suggested by Woodhead et al. (2019) for kimberlites along the western palaeo-margin of Pangea (i.e. southern Africa, Brazil, western Canada). The ancient age (> 1 Ga) of the subducted material rules out a direct genetic link between (deep) subduction and kimberlite formation. Considering that Woodhead et al. (2019) similarly modelled an old age for the subducted component in the source of kimberlites from southern Africa, Brazil and western Canada, it seems reasonable that Phanerozoic subduction could only have an indirect role, if any, in the genesis of all these kimberlites, including those from South Australia. A potential connection could be provided by the return flow from the lower mantle associated with substantial input of subducted material along the Pangea paleo-margins (Mather et al. 2023), with kimberlite (and UML) magmatism triggered by convective mantle upwelling. For the South Australian kimberlites and UML this mantle upwelling scenario is reinforced by their temporal and geographic overlap with the Karoo-Ferrar large igneous province (Burgess et al. 2015; Jiang et al. 2023; Ware et al. 2023) which also produced ~ 183 Ma ultramafic lamprophyres in Antarctica (Riley et al. 2003). The South Australian kimberlites and UML could be genetically related to magmatic activity at the periphery of the plume.

## Conclusions

We have presented new petrographic, geochemical and isotopic data on UMLs from the AFB and Gawler Craton in South Australia. Some of these bodies traditionally identified as kimberlites (Terowie, Eurelia and Angaston) have been reclassified as UMLs based on petrographic and geochemical analyses, combined with a review of available mica and spinel compositional data. The new isotopic data clearly demonstrate the substantial influence of crustal contamination on not only whole-rock Sr isotope compositions (a common trait for these rock types) but also Nd-Hf systematics of volcaniclastic kimberlite samples from the Gawler Craton. Conversely, the hypabyssal samples from Cleve (Gawler) and AFB display more homogenous isotopic compositions which provide direct petrogenetic information.

On the question of why kimberlites and UMLs coexist on the AFB, but only kimberlites are found on the Gawler Craton, we found no clear evidence linking lithospheric thickness to the genesis of one type over another, although the available paleogeotherm data is quite limited and fraught with large uncertainties. Similarly, the Nd-Hf isotopic data are inconclusive towards establishing systematic variations in their asthenospheric sources. We propose that it is the differences in the nature of the SCLM that is traversed and partly assimilated which may govern the type of magmatism expressed at the surface, whereby metasomatised lithologies are more common beneath Proterozoic mobile belts like the AFB relative to the cores of Archean cratons. Variations in mica concentrations reflected by bulk-sample K_2_O concentrations in kimberlites and UMLs reflect the variable extent of interaction with mica-bearing metasomatised lithosphere and lend support to this hypothesis. In this scenario UML melts may be generated upon the interaction of carbonate-rich asthenospheric melts with the more geochemically enriched lithospheric mantle material beneath the AFB.

Finally, we observe a notable temporal trend in Nd isotope composition in kimberlites and UMLs across the AFB region which may be due to a progressive incorporation of small amounts of subducted material into their mantle source over time. The ancient age (> 1 Ga) of the subducted component provided by mixing modelling suggest that subduction along the southern palaeo-margin of Pangea did not play a direct role, but perhaps facilitated the return flow of lower mantle material associated with the Karoo-Ferrar large igneous province. A similar scenario might apply to other kimberlite occurrences along the western palaeo-margin of Pangea even though a link to deep mantle upwelling is less clear.

## Supplementary Information

Below is the link to the electronic supplementary material.

Supplementary file1 (XLSX 1012 KB)

Supplementary file2 (PDF 802 KB)

## Data Availability

Data for this manuscript is included as supplementary material below and can be accessed freely at Earthbank by AuScope (see Boone et al., 2022) via the URL: 10.58024/AGUMFBAAFBE4.
