# Supplementary material for: New geochemical and Sr-Nd-Hf isotopic constraints on the genesis of kimberlites and ultramafic lamprophyres from the Adelaide Fold Belt and Gawler Craton, South Australia
Source: Mineral Petrol. 2025 Jul 9;119(4):1043–68. doi: 10.1007/s00710-025-00938-w (PMC12672780; doi:10.1007/s00710-025-00938-w)
Supplement: Supplementary file 2 — Supplementary file2 (PDF 802 KB) [file 710_2025_938_MOESM2_ESM.pdf]

SUPPLEMENTARY MATERIAL: New geochemical and Sr-Nd-Hf isotopic constraints on the genesis of kimberlites and ultramafic lamprophyres from the Adelaide Fold Belt and Gawler Craton, South Australia

Hayden Dalton[1], Andrea Giuliani[2, 3], Angus Fitzpayne[3], Bradley J. Peters[3]

🖂 Hayden Dalton

hayden.dalton@unimelb.edu.au

[1] School of Geography, Earth and Atmospheric Sciences, The University of Melbourne, Parkville, 3010 Victoria, Australia

[2] Earth and Planets Laboratory, Carnegie Institution for Science, Washington 20015 DC, United States

[3] Institute of Geochemistry and Petrology, Department of Earth and Planetary Sciences, ETH Zurich, Zurich 8092, Switzerland

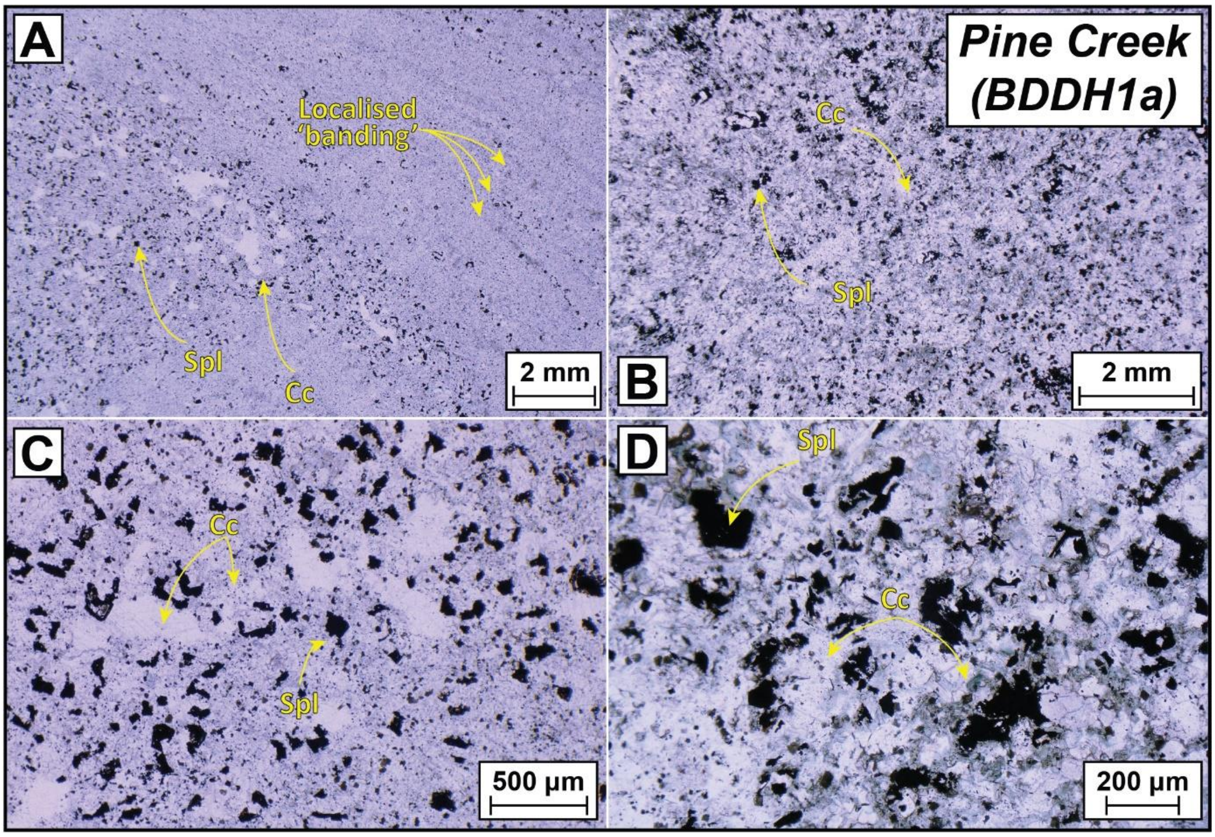

Supplementary Fig. S1 Representative plane-polarised light photomicrographs for Pine Creek sample BDDH1a, a spinel-bearing carbonatite. Mineral abbreviations are as follows, Spl: spinel; Cc: calcite. See main text for additional descriptions.

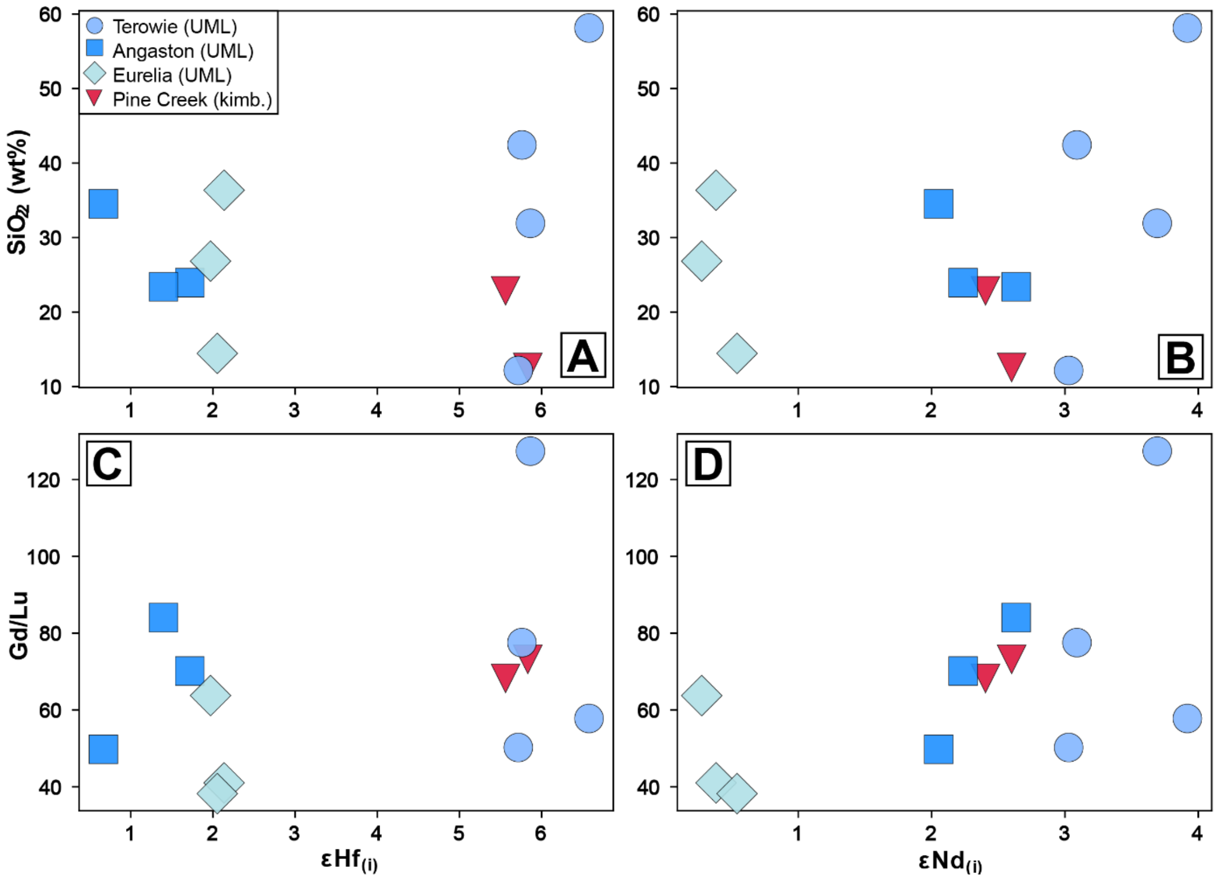

Supplementary Fig. S2 Co-variation charts of Nd-Hf isotope vs bulk-rock composition for Adelaide Fold Belt samples: A) εHf(i) vs SiO2; B) εNd(i) vs SiO2; C) εHf(i) vs Gd/Lu and D) εNd(i) vs Gd/Lu. Note that relative to Figure 10 (main text), no relationship is apparent in any of the co-variation charts.

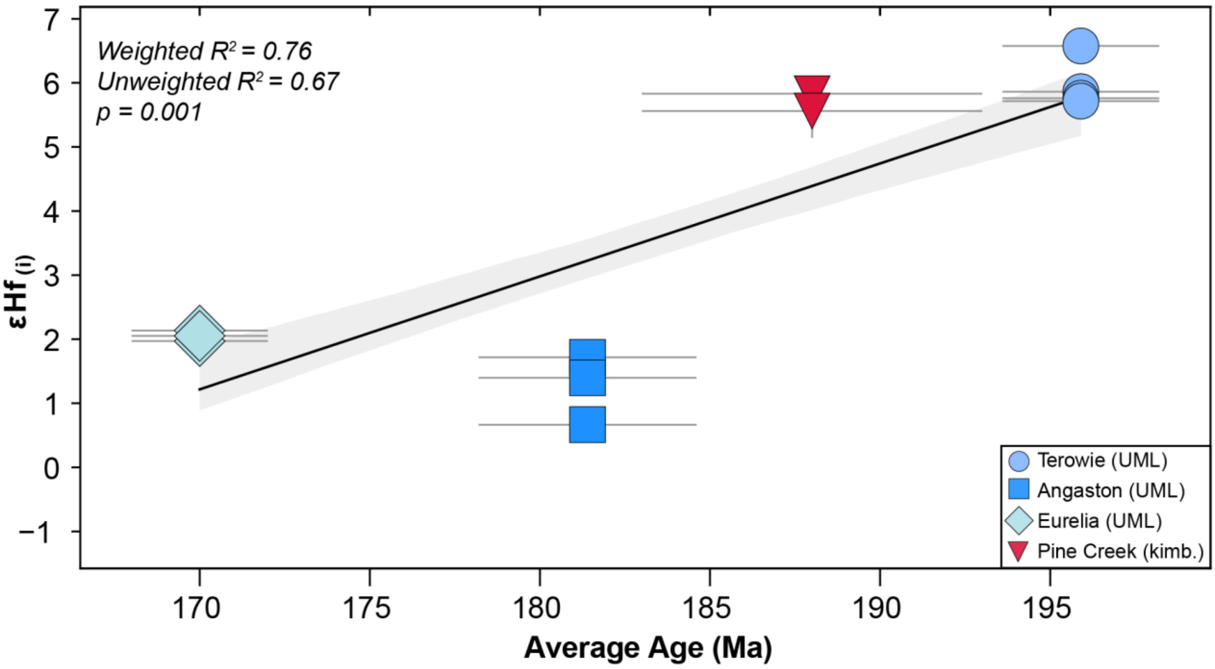

Supplementary Fig. S3 $\varepsilon Hf_{(i)}$ isotope compositions vs emplacement age chart for kimberlite and ultramafic lamprophyre (UML) for localities from the Adelaide Fold Belt only. R^2 is the correlation coefficient, where both an unweighted and weighted value, based on the uncertainty in individual data points, is shown. The shaded field represents the 2 standard deviation uncertainty envelope for the regression line.

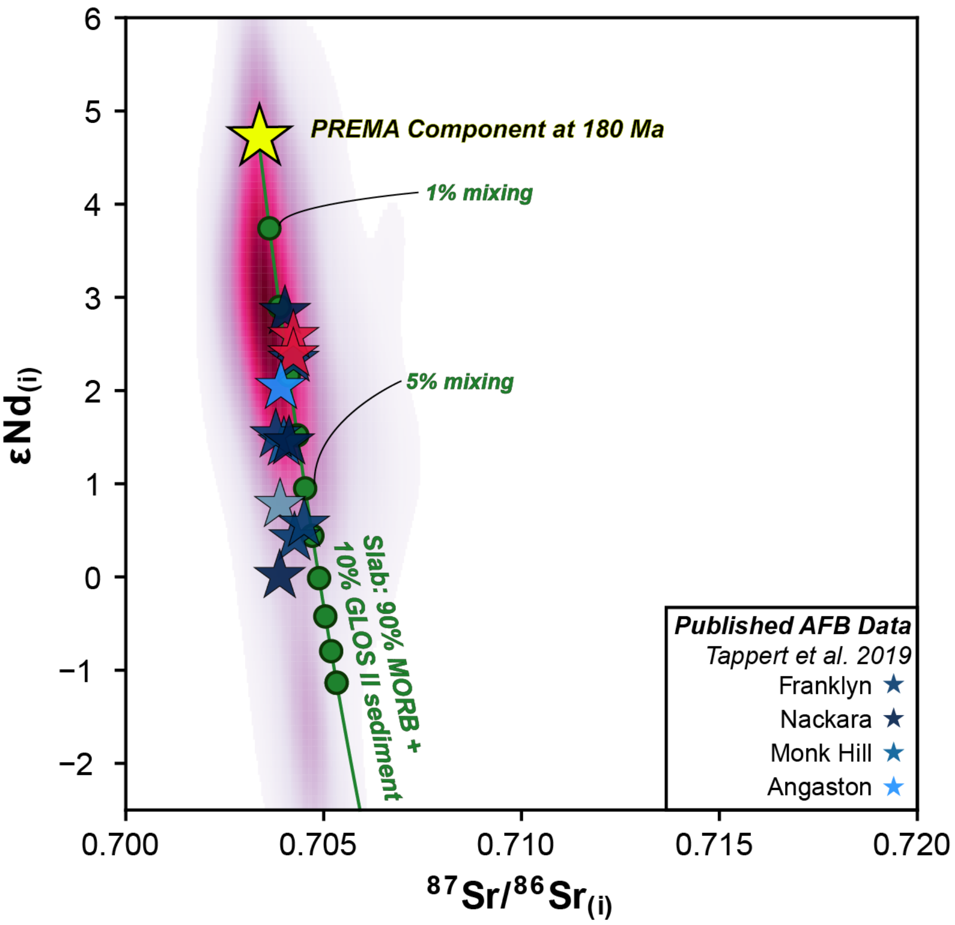

Supplementary Fig. S4 $^{87}Sr/^{86}Sr_{(i)}$ vs $\varepsilon Nd_{(i)}$ radiogenic isotope co-variation chart for Adelaide Fold Belt localities showing binary mixing relationship between a geochemically depleted asthenospheric PREMA component (Giuliani et al. 2021) and slab material subducted at 2.5 Ga. Subducted material is composed of 90% E-MORB (Gale et al. 2013) and 10% sediment, represented by either GLOSS II of Plank (2014) (Slab I). Compositions of subducted components adjusted to account for subduction-induced, sediment-fluid modification following Stracke et al. (2003). See Supplementary Table S6 for details of model parameters.
